# Occupancy as a key attribute linking saprotrophic fungi to soil carbon decomposition

**DOI:** 10.1093/nsr/nwag319

**Published:** 2026-05-28

**Authors:** Ziheng Peng, Yingyi Fu, Bernhard Schmid, Zhiheng Wang, Jiejun Qi, Yu Luo, Gehong Wei, Shuo Jiao

**Affiliations:** State Key Laboratory for Crop Stress Resistance and High-Efficiency Production, College of Natural Resources and Environment, Northwest A&F University, Yangling 712100, China; Shaanxi Key Laboratory of Agricultural and Environmental Microbiology, College of Life Sciences, Northwest A&F University, Yangling 712100, China; State Key Laboratory of Vegetation Structure, Function and Construction (VegLab), Ministry of Education Key Laboratory of Earth Surface Processes, College of Urban and Environmental Sciences, Peking University, Beijing 100871, China; Institute of Soil and Water Resources and Environmental Science, Zhejiang Provincial Key Laboratory of Agricultural Resources and Environment, Zhejiang University, Hangzhou 310058, China; Remotes Sensing Laboratories, Department of Geography, University of Zürich, Zürich 8057, Switzerland; State Key Laboratory of Vegetation Structure, Function and Construction (VegLab), Ministry of Education Key Laboratory of Earth Surface Processes, College of Urban and Environmental Sciences, Peking University, Beijing 100871, China; Shaanxi Key Laboratory of Agricultural and Environmental Microbiology, College of Life Sciences, Northwest A&F University, Yangling 712100, China; Institute of Soil and Water Resources and Environmental Science, Zhejiang Provincial Key Laboratory of Agricultural Resources and Environment, Zhejiang University, Hangzhou 310058, China; Shaanxi Key Laboratory of Agricultural and Environmental Microbiology, College of Life Sciences, Northwest A&F University, Yangling 712100, China; State Key Laboratory for Crop Stress Resistance and High-Efficiency Production, College of Natural Resources and Environment, Northwest A&F University, Yangling 712100, China; Shaanxi Key Laboratory of Agricultural and Environmental Microbiology, College of Life Sciences, Northwest A&F University, Yangling 712100, China

**Keywords:** occupancy, soil fungi, widespread species, ecological processes, carbon decomposition, geographic patterns

## Abstract

Understanding how fungal biogeography shapes ecosystem processes is central to predicting biogeochemical responses to environmental change. However, the properties of saprotrophic fungi that link their distribution patterns to belowground carbon cycling remain unclear. Here, we provide a comprehensive assessment linking widespread (high-occurrence) and narrowly distributed (low-occurrence) saprotrophic fungi (saprotrophs) to carbon decomposition processes across a broad latitudinal gradient from tropical to boreal zones. Our results show that the diversity of saprotrophic fungi is shaped by distinct community assembly processes, which in turn generate contrasting geographic patterns across latitudes. Widespread saprotrophs are mainly structured by large-scale climatic and local soil variables (environmental filtering) and show increasing diversity toward higher latitudes, whereas narrow-ranged saprotrophs are primarily constrained by the regional species pool (dispersal filtering), leading to declining diversity with latitude. By integrating large-scale biogeographic data with DNA stable isotope probing experiment, we provide evidence that fungal taxa actively participating in straw decomposition are dominated by widespread saprotrophs, underscoring their essential role in carbon cycling. Our findings highlight occupancy as a key biogeographic attribute regulating fungal contributions to carbon cycling and provide a predictive framework for understanding how belowground biodiversity shapes soil carbon dynamics under global change.

## Teaser

Not all soil fungi contribute equally to carbon cycling. Saprotrophic fungi with broad geographic occupancy contribute disproportionately to soil carbon decomposition across agricultural ecosystems, providing a predictive link between microbial biogeography and ecosystem function.

## INTRODUCTION

A major challenge in predicting ecosystem functions across spatial scales is understanding how ecological processes interact across different scales of space and time to shape biodiversity patterns and ecosystem functions [1]. While these relationships have been extensively studied in macroorganisms [2,3], their applicability to belowground microbial communities and associated biogeochemical cycles remain underexplored [4]. Although functional trait-based and genomic approaches have recently expanded microbial ecological studies [5], much of the literature still emphasizes taxonomic diversity and its responses to environmental drivers, leaving a critical gap in linking microbial biogeography to soil carbon cycling [6]. Addressing this gap is essential, given the critical role of microbes in global carbon reservoirs and their influence on climate feedbacks [7].

Soil saprotrophic fungi are among the most diverse and functionally important organisms on Earth [8], playing a central role in decomposition and carbon cycling [9–11]. However, not all saprotrophic fungi contribute equally to these processes [12,13]. Generalized processes, such as soil respiration and carbon decomposition, are expected to be more strongly shaped by widespread than by narrow-ranged taxa [14,15], not because narrow-ranged taxa lack the intrinsic ability to decompose carbon, but because widespread taxa occur more frequently and abundantly across soil environments. Moreover, the genetic and enzymatic capacities required for decomposition (e.g. carbohydrate-active enzymes and ligninolytic enzymes) are broadly shared among fungi and occur at high abundance across soil samples [16], thereby enabling widespread taxa to make a greater cumulative contribution at the community level. Nevertheless, some previous studies have overlooked the functional role of widespread species or have suggested that narrow-ranged species can also substantially contribute to these generalized processes [17,18]. These inconsistent findings highlight the need for a unifying metric that links species’ biogeographic patterns with their ecological roles.

**Figure 1.**
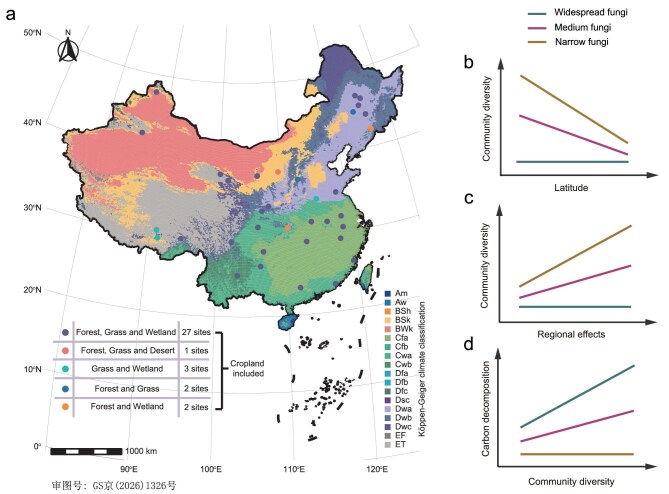
A conceptual diagram of how ecological processes shape geographical patterns of widespread and narrow-ranged saprotrophs and their contributions to carbon decomposition. (a) A map of 35 sites covers a wide range of climatic gradients. The color background represents the Köppen-Geiger climate classification (www.gloh2o.org/koppen). For geographic patterns (b), we hypothesize that widespread and narrow-ranged saprotrophs exhibit distinct latitudinal diversity patterns, with the diversity of narrow-ranged saprotrophs decreasing toward higher latitudes. For ecological processes (c), we propose that narrow-ranged saprotrophs are primarily structured by regional species pools, whereas widespread saprotrophs are shaped by large-scale climatic and local soil factors, leading to distinct diversity patterns. For soil carbon decomposition (d), we hypothesize that the distribution of widespread saprotrophs is mechanistically linked to variations in soil carbon decomposition.

Occupancy, defined as the proportion of sites in which a species occurs, provides a metric that captures the broad geographic distribution of taxa across regions [19]. Although not a classical morphological or physiological trait, occupancy can be considered as an emergent trait that integrates the combined outcomes of multiple underlying characteristics, including physiological tolerances, ecological strategies, and life-history features that collectively determine whether a species can persist across diverse environments [20,21]. Here, we propose a unifying framework that integrates microbial biogeography to ecosystem processes to examine how soil saprotrophic fungi (hereafter referred to as saprotrophs) with different occupancy levels contribute to carbon decomposition across broad climatic gradients (Fig. 1). Because occupancy reflects a species’ environmental breadth, demographic stability, and capacity to exploit diverse ecological niches, high-occupancy taxa are more likely to persist across heterogeneous landscapes [22]. Accordingly, we hypothesize that widespread saprotrophs will play a disproportionate role in soil carbon cycling since their broad distribution produces a more consistent functional presence across sites [23]; whereas narrow-ranged taxa, despite potentially strong local effects [24], have limited opportunities to influence processes at regional or continental scales. As broad-scale carbon dynamics require sustained microbial activity across diverse environments, they are expected to be driven primarily by fungal taxa with wide biogeographic distributions [25,26]. Yet, despite these theoretical expectations, the relative contributions of widespread and narrow-ranged saprotrophs to soil carbon decomposition across the terrestrial carbon pool remains poorly understood. Unraveling these mechanisms will provide critical insights into the link between microbial biogeography and carbon cycling, ultimately improving predictions of soil carbon dynamics under global environmental change.

In this study, we aimed to determine how widespread and narrow-ranged saprotrophs contribute differently to soil carbon decomposition, by linking their community diversity and geographic distribution patterns to decomposition processes. We conducted a large-scale soil survey of different terrestrial ecosystems across China, covering 847 soil samples collected from 35 provinces along a 3,400 km north-south and a 3,200 km east-west transect (Fig. 1a). Given that the species pools of plants and soil fungi generally exhibit clear latitudinal diversity gradients in natural ecosystems, with higher diversity toward the tropics [27–29], and that these species pools represent the broader reservoir of taxa that can potentially colonize and contribute to local community assembly [30,31], we hypothesized that this large-scale biogeographic pattern would also influence local saprotrophic fungal communities. Because local communities are assembled from regional species pools, broader regional diversity in tropical ecosystems should increase the likelihood of recruiting more taxa into local assemblages. We therefore expected local communities of narrow-ranged saprotrophs, which are more strongly constrained by regional species pools and dispersal limitation, to show a similar latitudinal pattern, with diversity increasing from the poles to the tropics (Fig. 1b and c). In contrast, widespread saprotrophs, due to their broader geographic distributions and greater environmental tolerance, may be less dependent on regional species pools and more closely linked to ecosystem functioning across sites. We therefore further examined whether variation in soil carbon decomposition is associated with the diversity of widespread saprotrophs (Fig. 1d). By integrating large-scale biogeographic surveys, functional experiments, and global datasets, our study provides empirical evidence linking microbial biogeography with ecosystem function and highlights fungal occupancy as a key trait connecting biodiversity patterns to soil carbon cycling.

## RESULTS

### General distribution of widespread and narrow-ranged saprotrophic fungi

Similar to other biological communities, saprotrophic fungal communities exhibit a skewed occupancy distribution, with relatively few high-occupancy species and many low-occupancy species (Fig. S1) [32,33]. We firstly grouped the soil fungi into widespread, medium, and narrow-ranged taxa according to occupancy. We divided amplicon sequence variants (ASVs), which we used as high-resolution ecological units to characterize fungal occupancy patterns rather than strict species definitions, by regional occurrence (the number of sites where an ASV taxon is present): widespread, >50% occurrence; medium, >20% and <40%; and narrow, <10% (Fig. S2). Most of the ASVs (89% of the total 5158 ASVs) had a narrow distribution, whereas only 67 ASVs had a widespread distribution. The medium-ranged fungi included 128 ASVs. The three different occupancy groups showed significant differences in their environmental ranges (the breadth of environmental conditions where an ASV is present) of mean annual temperature (MAT), mean annual precipitation (MAP), soil pH, and organic matter (OM), with environmental ranges decreasing as occupancy declined (Fig. S3). Moreover, we observed a strong correlation between occupancy and environmental range (Fig. S4) in all the occupancy groups. Given that occupancy reflects how broadly a species occurs across sites, we used it as a practical measure to characterize whether a saprotrophic fungal ASV is generalist or specialist in its habitat requirements [14]. The widespread, medium, and narrow-ranged groups showed differences in taxonomic composition (Fig. S2), in which the dominant phyla of widespread and medium-ranged saprotrophs were Ascomycota, Mortierellomycota, and Basidiomycota, while the narrow-ranged fungi were Ascomycota and Basidiomycota, followed by Mortierellomycota. Moreover, we found that the Ascomycota accounted for almost 60% of the widespread ASVs, indicating that the agricultural soil, consistent with a global-scale soil survey [34], was dominated by a small number of Ascomycota taxa.

### Contrasting biogeographic patterns of widespread and narrow-ranged saprotrophic fungi

**Figure 2.**
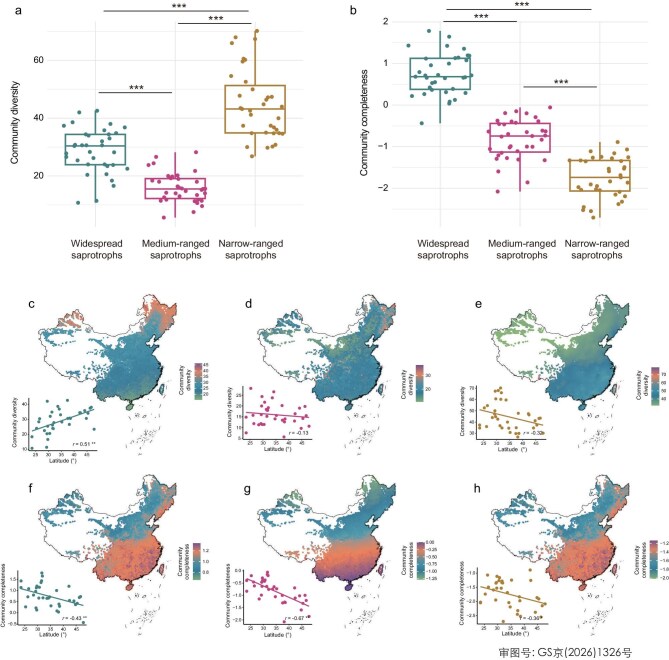
Community diversity, completeness and geographical patterns of widespread and narrow-ranged saprotrophs. Boxplot showing the community diversity (a) and community completeness (b) of saprotrophs with different occupancy. Spatial distributions of the community diversity (c–e) and community completeness (f–h) of different occupancy ASVs are mapped using the Cubist regression-based spatial interpolation method. Panels (c and f) represent widespread taxa, panels (d and g) represent medium-ranged taxa, and panels (e and h) represent narrow-ranged taxa. Ordinary least squares linear regressions were used to assess the relationships between diversity, completeness, and latitude. Statistical analysis was indicated by asterisks: *p < 0.05, **p < 0.01.

We further tested whether widespread, medium-, and narrow-ranged groups differed in geographic distribution and latitudinal patterns, potentially due to different assembly processes and environmental drivers. The narrow-ranged saprotrophs had the highest community diversity, while widespread and medium-ranged saprotrophs had less diverse communities (Fig. 2a). We found that community completeness decreased as occupancy declined, with narrow-ranged saprotrophs exhibiting the lowest community completeness (Fig. 2b). Community completeness reflects the proportion of species from the regional species pool that are observed in a local community, and is inversely related to dark diversity, with higher dark diversity corresponding to lower completeness. This pattern suggests that narrow-ranged saprotrophs were more strongly filtered of the local community than medium-ranged and widespread taxa during agricultural land use change.

As expected, we found that the latitudinal patterns of community diversity were significantly influenced by occupancy (latitude × occupancy category interaction: χ^2^ = 8.04, df = 2, *P* < 0.001). The community diversity of narrow-ranged saprotrophs showed the expected, yet not significant, trend of decreasing diversity with latitude (Fig. 2c–e). The community diversity of widespread saprotrophs, however, significantly increased with latitude, while medium-ranged saprotrophs were not statistically significant with latitude. These different latitudinal diversity patterns suggested that they may have resulted from different community assembly processes. The community completeness of all groups of saprotrophs significantly decreased with latitude (Fig. 2f–h), indicating that dark diversity increased with latitude. This suggests that harsher or more variable environmental conditions at higher latitudes may limit fungal establishment despite the presence of suitable species in the regional pool.

### Divergent community-assembly processes between widespread and narrow-ranged saprotrophic fungi

**Figure 3.**
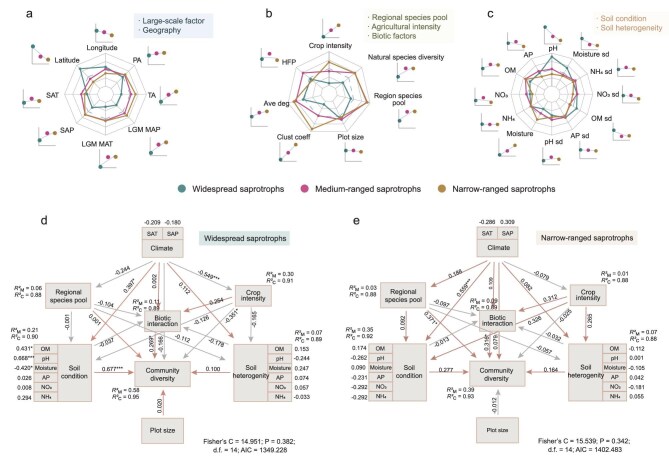
Relationships between environmental factors and community diversity of widespread and narrow-ranged saprotrophs. (a–c) Radar showing the Pearson correlation coefficients between environmental factors and community diversity of widespread (green), medium-ranged (red) and narrow-ranged (yellow) saprotrophs. The first circle from the outermost to the innermost indicates that the correlation coefficient is 1, the second circle indicates 0.5, the third circle (blue dotted lines) is 0, the fourth circle is −0.5, and the fifth circle is −1. The scatterplot and fitted line showing the variation of the correlation coefficient along widespread, medium-, and narrow-ranged taxa. (d and e) Structural equation models indicating possibly causal relationships between large-scale climate, crop intensity, soil condition, soil heterogeneity, biotic interactions, plot size, the regional species pool, and community diversity of widespread (d) and narrow-ranged (e) saprotrophs. Red and grey arrows indicate positive and negative relationships, respectively. Numbers adjacent to arrows are path coefficients (partial regression) which represent the directly standardized effect size of the relationship. The conditional (C) and marginal (M) *R*^2^ represent the proportion of variance explained by all predictors without and with accounting for random effects of ‘sites’. Significance levels of each predictor are *p < 0.05, **p < 0.01, ***p < 0.001. TA, temperature anomaly; PA, precipitation anomaly; SAT, summer temperature; SAP, summer precipitation; LGM MAT, mean annual temperature in the LGM; LGM MAP, mean annual precipitation in the LGM; HFP, human footprint pressure; Ave deg, average degree; Clust coeff, clustering coefficient. A list of environmental factors could be found in Table S1.

To explain diversity patterns of widespread and narrow-ranged saprotrophs within local communities, we tested associations between their diversity, completeness and underlying drivers, including geography, large-scale environmental variables (climate legacy and contemporary climate), regional effects (regional species pool and natural species diversity), agricultural intensity (human footprint pressure and crop intensity), local community assembly mechanisms (soil properties, environmental heterogeneity and biotic interactions), and plot size (Fig. 3a–c and Table S1). We found that the relationships between community diversity and most large-scale, regional, and local drivers differed between widespread and narrow-ranged saprotrophs (Fig. 3a–c). For example, the diversity of widespread saprotrophs increased with soil pH, while that of narrow-ranged saprotrophs decreased (Fig. S5); MAP was negatively associated with the diversity of widespread saprotrophs, but positively associated with that of narrow-ranged saprotrophs (Fig. S6). Moreover, the associations of environmental variables with the three different groups of saprotrophs were directional, that is, the strength of correlation between medium-ranged saprotrophs and environmental factors was between that of widespread and narrow-ranged groups. Contrary results were observed for community completeness where widespread and narrow-ranged saprotrophs showed similar relationships with most environmental drivers (Fig. S7a).

**Figure 4.**
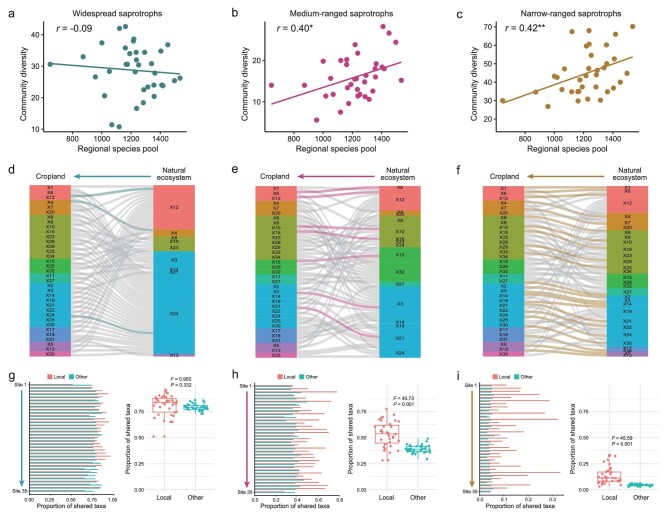
Linking regional species pool to community diversity of widespread and narrow-ranged saprotrophs. (a–c) The relationships between community diversity of saprotrophs with different occupancy and regional species pool. Ordinary least squares (OLS) linear regressions were used to assess the relationships between diversity and regional species pool. Statistical significance is indicated by asterisks: **p < 0.01. (d–f) Association of croplands in each region with shared saprotrophic fungal ASVs in natural ecosystems for widespread (left), medium-ranged (middle), and narrow-ranged (right) saprotrophs. Only the links between two natural ecosystems with the highest proportion of shared saprotrophic fungal ASVs and corresponding cropland in each region are shown here (2 lines for each cropland × 35 sites = 70 lines). When two natural ecosystems with the highest proportion of shared saprotrophic fungal ASVs with cropland in each region include the natural ecosystem in the same region, the links is given color. (g–i) The histogram shows the proportion of saprotrophic fungal ASVs shared by the cropland with the natural ecosystems in this region (local; red) and the natural ecosystems in other sites (other; green) for widespread (left), medium-ranged (middle) and narrow-ranged (right) saprotrophs. The boxplots show the difference between local and other across all sites.

Moreover, to explore potential mechanistic pathways linking saprotroph diversity to environmental drivers, we constructed structural equation models (SEMs) to evaluate hypothetical causal relationships between the diversity and completeness of widespread and narrow-ranged saprotrophs and underlying drivers (Fig. S8). We found that climate indirectly influenced the community diversity of widespread saprotrophs through its effects on cultivation intensity and soil variables, with soil pH and organic matter showing positive associations, whereas soil moisture and cultivation intensity exhibited negative associations (Fig. 3d and e). In contrast, the diversity of narrow-ranged saprotrophs was primarily shaped by the regional species pool and biotic interactions among species (Figs 3e and 4a). Precipitation, soil habitat filtering and heterogeneity jointly affected the community completeness of all three groups while regional species pool showed a minor effect on community completeness (Fig. S7b and c).

We compared the proportions of shared ASVs in croplands and surrounding natural ecosystems and found that the widespread saprotrophs shared the highest proportion of croplands and surrounding natural ecosystems only in three sites (Fig. 4a, d, left), the medium-ranged saprotrophs expanded to seven sites (Fig. 4b, e, middle), and the narrow-ranged saprotrophs expanded to 22 sites (Fig. 4c, f, right). This result indicated that narrow-ranged saprotrophs in the croplands are more closely related to surrounding natural ecosystems. We also compared the proportion of shared ASVs between croplands and surrounding natural ecosystems within sites and between croplands and natural ecosystems between sites (Fig. 4g–i). Widespread saprotrophs showed no significant difference between sites (*F* = 0.955, *P* = 0.332; Fig. 4g–i, left), while the proportion of shared narrow-ranged saprotrophs in croplands and surrounding natural ecosystems between sites was much lower than within sites (*F* = 45.59, *P* < 0.001; Fig. 4g–i, right). Our findings suggest that narrow-ranged saprotrophs are affected by regional drivers, whereas widespread saprotrophs are not.

To provide a general insight into the effects of regional species pool, we collected fungal ITS amplicon-based sequencing data at global scale to characterize the community assembly processes across the three different groups (Fig. S9). Our dataset covered agricultural systems and surrounding ecosystems, including forests, grasslands, deserts, shrublands, gardens, and wetlands. Consistent with our results at continental scale, global evidence indicated that the regional species pool is significantly negatively associated with the diversity of widespread (*r* = –0.26, *P* < 0.05) and medium-ranged saprotrophs (*r* = –0.16, *P* = 0.15), while is positively associated with the diversity of narrow-ranged saprotrophs (*r* = 0.89, *P* < 0.01; Fig. S9).

### Close linkage between widespread saprotrophic fungi and soil carbon decomposition potential

**Figure 5.**
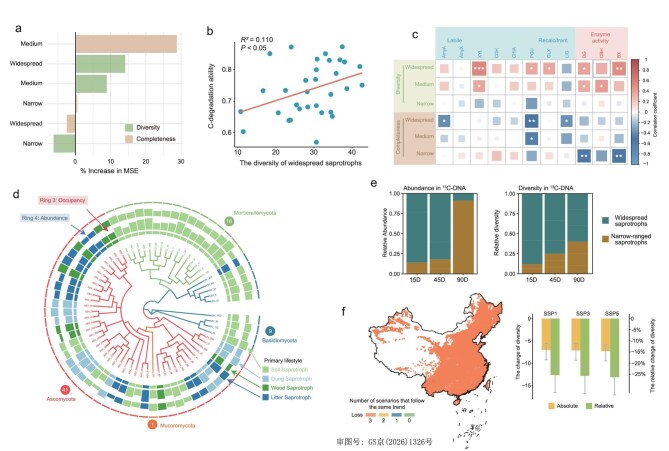
Relationships between widespread saprotrophs and soil carbon decomposition. (a) Variable importance metrics from random forest for the effects of diversity and completeness of saprotrophs with different occupancy on soil carbon decomposition. (b) The linear relationship between soil carbon decomposition and the diversity of widespread saprotrophs; statistical analysis was performed using ordinary least squares linear regressions. (c) Correlations between saprotrophic fungal diversity and completeness and soil carbon decomposition. The colour denotes the Pearson correlation coefficient. Significance levels are *p < 0.05, **p < 0.01, ***p < 0.001. (d) The phylogenetic relationships of widespread saprotrophic fungal ASVs (the innermost ring). The second ring represent fungal functional groups, as determined by FungalTraits. The third ring indicates occupancy and the fourth ring indicates relative abundance of widespread saprotrophic fungal ASVs. The colour of the outermost circle corresponds to the phylum level. (e) DNA-SIP experiment reveals fungal groups involved in carbon decomposition over time. 15D, 15 days; 45D, 45 days; 90D, 90 days. (f) Agreement with change in diversity of widespread saprotrophs across the different scenarios considered (‘loss’ reflects areas in which loss is predicted). Absolute and relative change for widespread saprotrophic fungal diversity assessed for scenarios SSP1-RCP2.6, SSP3-RCP7 and SSP5-RCP8.5. The bars and bar plots indicate the interquartile interval and median value for each scenario, respectively.

An important follow-up question is to determine which group—widespread or narrow-ranged saprotrophs—is more strongly associated with soil carbon decomposition. Our results showed that the community diversity of widespread saprotrophs was a major factor contributing to carbon decomposition potential (Fig. 5a), showing a significant positive correlation (*r* = 0.37, *P* < 0.05) (Fig. 5b). Specifically, the community diversity of widespread saprotrophs had significant positive correlations with the abundance of C-degradation genes (*xly, pgu*, and *glx*) and C-degradation enzymes (BG, CBH, and BX) (Fig. 5c), indicating their potential functional capacity for carbon decomposition. No significant relationship between the community diversity of narrow-ranged saprotrophs and these C-degradation indicators was observed. It is important to clarify that these indicators reflect the decomposition potential rather than direct measurements of decomposition rates in the field. The 67 widespread saprotrophic fungal taxa spanned dung-, litter-, soil-, and wood-associated guilds (Fig. 5d). Their broad distribution in agricultural soils highlights their central role in sustaining carbon turnover across multiple substrate types. However, the stronger correlations between widespread saprotrophs and decomposition-related indicators could simply reflect higher detectability and abundance in amplicon datasets rather than true functional dominance.

To address this, we conducted a DNA stable isotope probing (DNA-SIP) experiment to determine whether the fungal taxa actively participating in straw decomposition were predominantly widespread or narrow-ranged saprotrophs. During the decomposition of maize straw, the ^13^C-labeled DNA originated predominantly from widespread saprotrophic fungi (Fig. 5e), confirming their role as the dominant active decomposers. Notably, this functional dominance was observed even though narrow-ranged fungi exhibited higher local taxonomic diversity across the 35 surveyed sites. The DNA-SIP results thus verify that the stronger correlations between widespread saprotrophs and decomposition-related indicators reflect a genuine, dominant functional role in carbon turnover.

Given the close linkage between widespread saprotrophs and carbon decomposition potential, we projected changes in their diversity under global change scenarios (RCP2.6–SSP1, RCP7.0–SSP3 and RCP8.5–SSP5 up to 2100). Our analysis showed a 25% decrease in the community diversity of widespread saprotrophs in most sites of China regardless of the climate scenarios considered (Fig. 5f). These results implied that temperature-driven reductions in the community diversity of widespread saprotrophs might decrease carbon decomposition and promote carbon sequestration in agricultural soils under projected climate change.

## DISCUSSION

A major challenge in ecological research is conducting empirical studies to unravel large-scale patterns of biodiversity [35,36]. Large-scale ecological drivers, regional species pool and local assembly processes are recognized as the drivers of large-scale spatial variation in diversity patterns in the framework of community assembly [37]. However, not all species are equally affected by these drivers. Our study contributes to this field by demonstrating that community assembly processes in soil saprotrophic fungi differ according to occupancy, with direct implications for soil carbon cycling. Narrow-ranged taxa were primarily structured by regional species pools, whereas widespread taxa responded mainly to large-scale climatic and edaphic factors. These contrasting assembly mechanisms explain why narrow-ranged saprotrophs show latitudinal diversity patterns closely tied to regional species pools, while widespread saprotrophs maintain distributions across broad gradients. Importantly, by integrating large-scale surveys with isotope-probing experiments, we found that widespread taxa were more strongly associated with indicators of soil carbon decomposition potential than narrow-ranged taxa. These results suggest that widespread saprotrophs may contribute more consistently to potential decomposition activity across sites, although they do not demonstrate direct effects on realized carbon losses from soils. Consequently, temperature-driven declines in widespread saprotrophs could plausibly reduce the capacity for microbial carbon transformation, with potential implications for soil organic matter dynamics and soil fertility. Our study highlights occupancy as a key trait that integrates ecological processes across scales and connects biodiversity to carbon cycling.

Not all species are found everywhere, which is one of the fundamental concepts of ecology [38]. A species’ occupancy reflects underlying functional and life-history traits such as metabolic breadth, enzyme repertoires, stress tolerance and dispersal capacity, which together influence persistence and spread across environments [14]. Recent trait-based studies show that fungal network and physiological traits (e.g. hyphal network architecture, enzymatic capacity) are predictive of ecological strategies and biogeographic patterns [39–41], supporting the view that traits can help explain why some fungi are widespread while others remain narrow-ranged. Importantly, occupancy itself can also be regarded as an emergent trait, reflecting the integration of the net outcome of multiple physiological, morphological and life-history characteristics that determine ecological strategies and adaptive capacity [20,21]. A global metacommunity study revealed that anthropogenic pressures favor widespread species while disadvantaging narrow-ranged taxa over time [42]. In our study, we observed contrasting relationships between saprotrophic fungal diversity and environmental drivers for widespread and narrow-ranged saprotrophs, highlighting the role of occupancy in community assembly. A subset of fungi can exist across a wide range of environmental conditions and are more likely to be widespread; they disperse over long distances, establish new populations, and proliferate [43,44] being less constrained by extrinsic factors such as microhabitat and soil conditions. In contrast, narrow-ranged saprotrophs persist only under specific environmental conditions and are often endemic. Their distribution is constrained by climatic tolerance and primarily shaped by regional and local processes, including soil moisture and nutrient heterogeneity [40,45]. The SEMs also fitted our hypothesis, showing that diversity of narrow-ranged saprotrophs was influenced by the regional species pool and local assembly processes, whereas diversity of widespread saprotrophs was more strongly associated with large-scale environmental variation such as climate and soil pH.

Agricultural intensification and expansion is the primary driver of terrestrial biodiversity loss throughout the 21st century [46], and this risk will further increase with growing human populations [47]. Land use change-driven declines of soil biodiversity are largely attributable to the loss of narrow-ranged species [48,49], which often have more specific environmental and resource requirements and are therefore more vulnerable to habitat alteration caused by nutrient enrichment, irrigation management, and shifts in aboveground vegetation. Although ecologists have long recognized that local communities are influenced by regional species pools [30], our results show that these species-pool effects do not influence widespread taxa, which harbor wider environmental adaptation and greater dispersal ability. While large-scale climate, soil properties, and soil heterogeneity produced broadly similar completeness patterns across widespread, medium-, and narrow-ranged taxa, the influence of the regional species pool differed markedly among occupancy groups [50,51]. Community completeness of narrow-ranged saprotrophs increased proportionally with species pool size, whereas widespread saprotrophs showed weaker sensitivity, indicating that agricultural soils are not saturated and can accommodate additional narrow-ranged taxa while widespread taxa are already prevalent across sites. Because narrow-ranged saprotrophs contribute nearly half of the total diversity of soil saprotrophic fungi, the strong dependence of their observed diversity and completeness on the regional species pool underscores the importance of maintaining regional species pools to safeguard belowground biodiversity in agricultural landscapes.

Saprotrophic fungi are among the most important soil decomposers on Earth and are also regarded as important drivers of the global carbon cycle [52]. By secreting diverse enzymes to degrade complex organic compounds such as cellulose, chitin, and soil organic matter, they regulate carbon fluxes from soils to the atmosphere [9]. Our results show that soil carbon decomposition is strongly associated with the diversity of widespread saprotrophs, rather than narrow-ranged saprotrophs, highlighting occupancy as a central trait linking community assembly to carbon cycling. Trait-based studies typically quantify potential functional capacities under controlled or standardized conditions, where constraints among traits can be more readily detected. However, in natural soil environments, resource acquisition is itself a prerequisite for maintaining broad environmental occupancy [53,54], as saprotrophic fungi must continuously exploit heterogeneous substrates to sustain their populations across diverse habitats. Consequently, environmental tolerance and resource acquisition may become mutually reinforcing rather than mutually exclusive in situ. Broadly distributed fungi may therefore maintain or upregulate key decomposition functions not despite their wide environmental niche, but because successful colonization across variable environments requires versatile metabolic machinery [22,55]. Future work integrating trait-based assessments with field-based functional measurements will be essential for determining when and where these trade-offs manifest in fungal ecology. An alternative explanation is that widespread taxa may simply reach higher local abundance, and their strong influence on decomposition could arise from numerical dominance rather than superior functional capacities. Both mechanisms could contribute to the observed patterns: widespread fungi may influence decomposition through their functional traits, their local abundance, or through the interaction of both factors.

By combining large-scale field surveys and functional experiments, our study provides empirical evidence that fungal occupancy strongly shapes soil carbon decomposition across environmental gradients. Furthermore, temperature-driven declines of widespread saprotrophs could substantially reduce potential contribution to carbon decomposition, slowing decomposition and potentially increasing soil carbon sequestration in croplands. These findings are consistent with empirical studies and a global-scale survey showing that temperature reduces soil fungal diversity, particularly fungal decomposers, which are positively correlated with ecosystem respiration [56,57]. Collectively, these results underscore that the distribution and traits of widespread fungal decomposers are critical determinants of ecosystem carbon dynamics and highlight the unique contribution of this work in linking saprotrophic fungal biogeography, functional traits, and global carbon cycling.

## CONCLUSIONS

**Figure 6.**
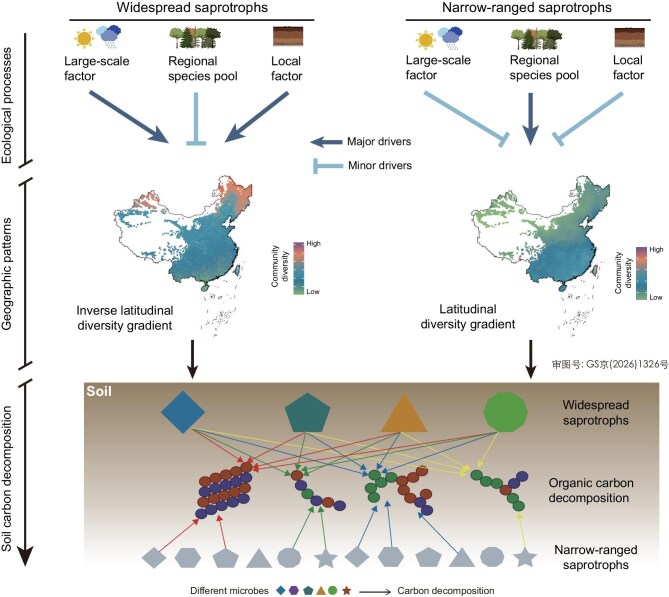
Conceptual model linking ecological processes to geographic patterns and soil carbon decomposition of widespread and narrow-ranged saprotrophs. Narrow-ranged saprotrophs are primarily structured by regional species pools, whereas widespread saprotrophs are shaped by large-scale climatic and local soil factors, leading to distinct diversity patterns. While widespread saprotrophs exhibit latitudinal diversity patterns, the diversity of narrow-ranged saprotrophs decreases toward higher latitudes, forming an inverse latitudinal diversity gradient. These contrasting distribution patterns further influence soil functions, as the distribution of widespread saprotrophs is mechanistically linked to variations in soil carbon decomposition.

There is growing interest how occupancy concepts may be used to predict within community diversity and thus ecological processes. By examining the geographic breadth of saprotrophic fungal taxa, we show how biogeographical history, large-scale environmental filtering, the regional species pool, local processes, and agricultural intensity collectively shape saprotrophic fungal community diversity. Furthermore, we compare widespread and narrow-ranged taxa to assess how these factors influence their respective roles in driving soil carbon decomposition in agricultural systems (Fig. 6). Our results reveal a general mechanism linking saprotrophic fungal occupancy to ecological processes. Specifically, the diversity of narrow-ranged saprotrophs is strongly shaped by the regional species pool, reflecting patterns also observed in natural ecosystems. This highlights the potential to enhance and restore agricultural fungal diversity through regional diversity conservation. In contrast, widespread saprotrophs are primarily structured by large-scale climatic factors and contribute disproportionately to soil carbon decomposition potential. A projected loss in the diversity of widespread saprotrophs under future climate scenarios may suppress soil carbon loss from croplands. By linking microbial occupancy with their functional roles, our study provides new insights into how biogeographic patterns govern key belowground processes in human-dominated ecosystems.

## METHODS

### Study design and fungal community characterization

Soil samples were collected from 169 maize fields across 35 sites spanning major climatic zones of China. Soil fungal communities were characterized using ITS amplicon sequencing on the Illumina MiSeq platform. Sequence processing, taxonomic assignment, and functional guild classification were conducted using standard bioinformatic pipelines. Detailed sampling procedures, DNA extraction, sequencing protocols, and bioinformatic analyses are provided in the Supplementary Methods.

### Environmental drivers and soil carbon decomposition potential

We evaluated multiple classes of predictors potentially shaping saprotrophic fungal diversity, including regional species pools, climate, soil properties, agricultural intensity, and biotic interactions. Soil carbon decomposition potential was assessed using carbon-degradation functional genes and extracellular enzyme activities. Detailed descriptions of predictor variables, network construction, qPCR assays, and enzyme measurements are provided in the Supplementary Methods.

### DNA stable isotope probing experiment

To determine whether widespread saprotrophic fungi actively participate in maize straw decomposition, we conducted a ^13^C-labeled maize straw incubation experiment and tracked substrate assimilation using DNA stable isotope probing (DNA-SIP). Heavy DNA fractions enriched with ^13^C were identified through density-gradient centrifugation and subsequently characterized by ITS amplicon sequencing. Active decomposer taxa were identified by comparing ^13^C-enriched and control communities, and were then matched to the nationwide fungal reference dataset to determine whether they belonged to widespread or narrow-ranged taxa. Detailed experimental procedures are provided in the Supplementary Methods.

### Occupancy, diversity and completeness

Saprotrophic fungal occupancy was quantified as the proportion of sites in which each ASV occurred. Widespread saprotrophs were defined as ASVs occurring in >50% of sites, whereas narrow-ranged saprotrophs occurred in <10% of sites. Community diversity was quantified as saprotrophic fungal richness at each site. Community completeness was estimated using dark diversity and calculated as the logarithm of observed richness relative to dark diversity. Dark diversity was estimated using the hypergeometric method implemented in the DarkDiv package. Spatial patterns of diversity and completeness were visualized using Cubist-based spatial interpolation. Detailed descriptions of occupancy classifications, dark diversity estimation, and spatial modelling are provided in the Supplementary Methods.

### Statistical analyses and future projections

We used Pearson correlation analyses to evaluate relationships among environmental variables, saprotrophic fungal diversity and completeness, and indicators of soil carbon decomposition. Structural equation modelling (SEM) was used to assess direct and indirect effects of environmental drivers and regional species pools on fungal diversity and completeness. Random forest models were applied to quantify the relative importance of diversity and completeness metrics in predicting soil carbon decomposition potential.

To assess potential future changes in widespread saprotrophic fungi, we developed predictive models based on the most important environmental drivers and projected diversity under future climate scenarios using CMIP6-derived climate datasets. In addition, a global meta-analysis based on published ITS sequencing datasets from agricultural and surrounding natural ecosystems was conducted to evaluate the generality of relationships between regional species pools and saprotroph occupancy patterns. Detailed descriptions of statistical analyses, model construction, future projections, and meta-analysis procedures are provided in the Supplementary Methods.

## Supplementary Material

nwag319_Supplemental_File

## Data Availability

The raw sequence data that support the findings of this study are openly available in the Beijing Institute of Genomics (BIG) Data Center, Chinese Academy of Sciences, under BioProject accession no. PRJCA020242 and are publicly accessible at http://bigd.big.ac.cn/gsa.
