# Supplementary material for: Occupancy as a key attribute linking saprotrophic fungi to soil carbon decomposition
Source: Natl Sci Rev. 2026 May 28;13(12):nwag319. doi: 10.1093/nsr/nwag319 (PMC13317454; doi:10.1093/nsr/nwag319)
Supplement: nwag319_Supplemental_File [file nwag319_supplemental_file.docx]

*Supplementary information for*

Occupancy as a key attribute linking saprotrophic fungi to soil carbon decomposition

Ziheng Peng et al.

Content of figure and table

Methods

Supplementary Table 1-2

Supplementary Fig. 1-13

**Supplementary Methods**

***Study design***

Soil samples were collected from 169 agricultural fields in 35 sites along a 3400 km latitudinal (23°39’ N to 47°79’ N) and 3200 km longitudinal gradient (86°30’E to 126°10’E). The distribution of fields among sites was uneven, with 30 sites containing five fields each, four sites containing four fields each, and one site containing three fields. Our sampling was conducted during the planting season of maize (*Zea mays*) in July and August 2019. At each field, soil samples were collected from the surface layer (0–20 cm) using three soil cores with a 5-cm-diameter auger and then thoroughly mixed to form one composite sample per field. These soil samples were sieved through a 2.0-mm mesh to remove plant roots, litter, rocks, and other debris. Each soil sample was divided into two subsamples where one was frozen at −80 °C for DNA extraction and microbial analysis and the other was air dried for measurement of soil physical and chemical properties. The study sites represented a wide range of environmental gradients of climate, soil, and vegetation types (from tropical to boreal zones, **Fig. 1a**). MAP and MAT in these sites ranged from 99 to 1775 mm and –2.8 to 21.9ºC, respectively, from 1970–2020. To minimize variation between fields, we only used fields planted with maize, one of the most frequently cultivated crops throughout China and the world [1].

***Amplicon sequencing and processing of soil fungi***

The diversity of soil fungi was measured by amplicon sequencing using an Illumina MiSeq platform. Genomic DNA was extracted from 0.5 g of the soil samples using the MP FastDNA spin kit for soil (MP Biomedicals, Solon, OH, USA) according to the manufacturer’s instructions. A portion of the ITS1 region of the universal fungal DNA barcode was sequenced using the ITS5-1737F (5’- GGAAGTAAAAGTCGTAACAAGG-3’) and ITS2-2043R (5’- GCTGCGTTCTTCATCGATGC-3’) primer sets on a Illumina MiSeq 300PE (Illumina Inc.). Bioinformatics processing, including filtering, dereplication, sample inference, chimera identification, and merging of paired-end reads, was performed using the Divisive Amplicon Denoising Algorithm 2 (DADA2), a model-based approach for correcting Illumina amplicon errors without constructing OTUs [2]. Compared to other methods, DADA2 can identify more real variants and output fewer spurious sequences [2]. The taxonomical annotation of the amplicon sequence variants (ASVs) was achieved with a naïve Bayesian classifier using the UNITE v. 10 database [3]. All bioinformatics processing was done in the dada2 package in R. The identification of saprotrophic fungi was conducted using the FungalTraits database [4]. In total, we identified 38,111 fungal ASVs from soil samples collected across 169 maize fields at 35 sites, of which 5,158 were classified as saprotrophic fungi.

***Environmental drivers***

***Regional species pool and natual species diversity***

To evaluate the regional species pool, we collected soil samples from natural ecosystems in each location. These natural ecosystems were the dominant types within 2 km of a maize field to ensure that the natural systems and agricultural systems experienced similar environmental condition (climate and soil type), covering the four major ecosystems types forest, grassland, wetland, and desert (**Fig. 1a**). A total of 678 soil samples were collected. These soil samples were sieved through a 2.0-mm mesh to remove plant roots, litter, rocks, and other debris and then measured for soil fungi. The regional species pool was calculated as total saprotrophic fungal richness of all ecosystems, including the target agricultural fields (n = 169), at each site (from 19 to 26 samples per site; 678+169 = 847 in total). For each focal site, the number of analyzed fields used to estimate the regional species pool ranged from 19 to 26, reflecting variation in the availability of surrounding ecosystems and sampling effort among regions. To evaluate whether differences in field number influenced estimates of regional species pool richness, we tested the relationship between field number and regional species pool size and found no significant association (*r* = -0.12, *p* = 0.49; Supplementary Fig. 11). In addition, regional species pool richness standardized by the number of sampled fields per site was strongly positively correlated with the original estimates (*r* = 0.88; Supplementary Fig. 11), indicating that our results were robust to differences in sampling effort. Natual species diversity was quantified as the total saprotrophic fungal richness in all natural ecosystems.

***Large-scale environmental factors***

Temperature and precipitation are important predictors of fungal biodiversity patterns at the global scale [5, 6]. We extracted MAT, MAP, monthly temperature at sampling time (July and August, i.e. summer temperature) and monthly precipitation at sampling time (summer precipitation) with a spatial resolution of 2.5 minutes (~ 4.5 km) from the WorldClim database (<https://www.worldclim.org/>). We used 2.5 minutes grid resolution rather than 30 arc seconds (~ 1 km) because climatic variables were used to characterize conditions within the region. These variables represented contemporary climatic conditions.

The legacy impacts of past climates are important drivers of plant and soil bacterial communities [7, 8]. We obtained MAT and MAP values near the last glacial maximum (LGM, ~22’000 years before present) from the CHELSA project [9]. CHELSA-TraCE21k data provide climatic variables at 30 arcsec spatial resolution in 100-year time steps for the last 21,000 years [10]. We calculated temperature anomaly (TA) and precipitation anomaly (PA) as the absolute values of the differences in MAT and MAP between 21’000 years ago (near LGM) and now to represent past climate change.

***Soil properties and heterogeneity***

Soil properties are important predictors of local fungal community composition [11]. We selected six soil variables to represent soil properties, namely soil pH, organic matter (OM), soil moisture (SM), available phosphorus (AP), and two forms of available nitrogen (NO_3_-N and NH_4_-N). These indicators have also been suggested as the main soil variables influencing bacterial diversity patterns at global and regional scales [12-14]. Soil pH was assessed in a 1:5 suspension (soil to distilled water) using a pH meter. Organic matter was determined calorimetrically following oxidation with a combination of potassium dichromate and sulfuric acid. Soil moisture was measured by the gravimetric method after samples were oven-dried at 100 °C for 24 h. NO_3_-N and NH_4_-N concentrations were measured using 1M KCl solution with a continuous-flow auto analyzer (AA3, SEAL Analytical). Available phosphorus concentrations were extracted using NaHCO_3_ and measured by molybdenum blue colorimetry. We determined soil physicochemical properties for each of the 169 maize fields. Then local soil condition was calculated as the average of all plots within agricultural sites for each soil property. Local soil heterogeneity was calculated as the standard deviation of the six soil variables within agricultural sites.

***Agricultural intensity***

Agricultural management intensity is one of the major drivers of soil biodiversity in croplands [15, 16]. We used crop intensity and human footprint pressure to represent agricultural management intensity. Crop intensity was defined as the number of crop planting and harvesting cycles per year from the GCI30 dataset [17]. This dataset provides a 30-m resolution cropping intensity dataset with global extent. The values of cropping intensity are 1, 2, or 3 representing single cropping, double cropping, or triple cropping, respectively. Human footprint pressure contained eight variables measuring direct and indirect human pressures, namely (1) percent of built environments; (2) percent crop land; (3) percent pasture land; (4) human population density; (5) night-time lights; (6) railways; (7) roads; and (8) navigable waterways [18]. These variables were weighted according to estimates of their relative levels of human pressure and then summed to create the standardized human footprint.

***Biotic interactions***

Biotic interactions are fundamental drivers governing biodiversity locally [19, 20]. Species association is regularly used as a proxy for biotic interaction in ecology and biogeography [21, 22]. One way to infer species association is based on species co-occurrence. Here, we conducted co-occurrence networks for the maize fields at each site and mapped the imprints of biotic interactions on the assembly of soil saprotrophic fungi. The association network examined the pairwise correlation coefficients of species based on saprotrophic fungal ASV abundance data. Robust correlations based on Spearman's correlation coefficients (*ρ*) of >.6 or <−.6 and false discovery rate-corrected *p*-values < .001 were used to construct networks. The average degree and average clustering coefficient were regarded as biotic factors in examining their contribution to diversity patterns [23]. The average degree referred to the average edges of each node, representing species connectivity in the community. The average clustering coefficient measured how well nodes related to their neighbors.

***Soil carbon decomposition potential***

To assess the potential for soil carbon decomposition, we measured functional gene (*amyA, amyX, cdh, chiA, glx, lig, pgu* and *xylA*) and extracellular enzyme activities (β-glucosidase (BG), cellobiohydrolase (CBH), β-xylosidase (BX)) [24, 25]. First, qPCR analyses were conducted to detect the absolute abundance of the functional genes [26]. Each primer set was conducted in triplicate in qPCR reactions at a threshold cycle (CT) of 31 as the detection limit. The initial enzyme activation was measured at 95 °C for 5 min, followed by 40 cycles of denaturation at 95 °C for 30 s, annealing at 58 °C for 30 s and extension at 72 °C for 30 s. Detailed information is provided in Supplementary Table 2. Second, the activities of the extracellular enzymes were measured fluorometrically following using a 200 μM solution of substrates labeled with 4-methylumbelliferone (MUB) or 7-amino-4-methylcoumarin (AMC). After a 4 h incubation at 20 °C, plates were centrifuged, and the supernatant was transferred to black, flat-bottom 96-well plates. Fluorescence was measured using a microplate reader with 365 nm excitation and 450 nm emission filters. Although these metrics do not quantify realized carbon mineralization rates or carbon fluxes directly, they are widely used proxies of decomposition capacity potential [27], capturing the dominant hydrolytic pathways involved in soil organic matter breakdown [28].

**DNA stable isotope probing experiment**

An incubation experiment over 90 days was conducted to investigate whether the decomposition of maize straw was supported by the widespread saprotrophs. Maize straw (99 atom %, Sigma-Aldrich Co., MO, USA) was added at 1000 mg C kg^-1^ soil (o.d.) into 10 g soil with three replicates. These were then put into glass jars, sealed with a rubber bung, and incubated in a randomized block design at 25 ºC in the darkroom. Soils were destructively sampled at days 15, 45 and 90.

DNA extraction was performed using a FastDNA Spin Kit according to the DNA protocol (MP Biomedicals, Santa Ana, CA, USA). We performed the isopycnic gradient centrifugation to separate the “heavy fractions” (^13^C-labeled DNA) from total DNA (Lueders et al., 2004). Briefly, approximately 3 μg of extracted DNA were mixed with cesium chloride (CsCl) to obtain an original density of 1.725 g mL^-1^. The mixture was centrifuged at 177,000 x g for 44 h at 20°C. After centrifugation, the gradient medium was replaced with sterile water from the top of the ultracentrifuge tube at a controlled flow rate of 0.34 ml min^-1^ using a NE-1000 single syringe pump (New Era Pump Systems Inc., Farmingdale, NY, USA). Following this step, 340 μl of each DNA gradient fraction was generated and the buoyant density of each component was measured using an AR200 digital handheld refractometer (Reichert, Inc., Buffalo, NY, USA). Each DNA fraction was assayed on an iCycleriQ 5 thermal cycler (Roche Diagnostics, Meylan, France) using the primer pair ITS5-1737F and ITS2-2043R to determine the copy number of fungal genes. Each reaction was performed in a 20 μl volume (10 μl of SYBR Premix Ex Taq (TaKaRa Biotechnology, Otsu, Shiga, Japan), 0.4 μM of each primer and 1 μl of DNA template (1-10 ng). Thermal cycling conditions were: initial denaturation at 95 °C for 180 s, 25 cycles of denaturation at 98 °C for 20 s, annealing at 55 °C for 15 s, extension at 72 °C for 15 s, and final extension at 72 °C for 1 min.

Selected DNA samples were prepared for Illumina MiSeq sequencing. The fungal gene fragments were amplified using barcodes and index primers ITS5-1737F and ITS2-2043R, and the products were prepared and sequenced on the Illumina Miseq platform operated by the Majorbio platform (Illumina, San Diego, USA). Pooled PCR amplicons from triplicate reactions were purified by using the QIAquick PCR Purification Kit (Qiagen, Shenzhen, China) and quantified by using a Nanodrop ND-1000 spectrophotometer (Thermo Scientific, Waltham, MA, USA). Amplicons from all samples were combined in equal masses. The purified mixture was diluted and denatured according to the Illumina MiSeq kit preparation guidelines to obtain an 8 pmol l-1 amplicon library and mixed with an equal volume of 8 pmol l-1 PhiX (Illumina). Finally, 600 μl of mixture amplicons were loaded with read-1, read-2 and indexed with sequencing primers, and paired-end sequencing (250 bp each) was done on the Illumina MiSeq platform. ^13^C-labeled ASVs (log2-fold>1, *P*<0.05) were filtered by comparing reads between ^13^C-treated and ^12^C-treated samples using DESeq2 (Supplementary Fig. 12). To determine whether saprotrophic fungi actively involved in maize straw decomposition corresponded to previously defined widespread or narrow-ranged taxa, these sequences were then compared against the national-scale ITS reference library, in which fungi had been previously classified as widespread or narrow-ranged based on occupancy across sites. Sequence matching was performed using BLAST (version 2.15.0) with a strict identity threshold of 100%, allowing assignment of the ^13^C-labeled fungi to the corresponding regional category. This approach enabled us to link the fungi actively assimilating maize-derived carbon to their broad-scale distribution patterns.

***Identification of saprotrophic fungal occupancy***

The identification of widespread and narrow-ranged saprotrophs was conducted at ASV level. We estimated species occupancy as area of occupancy (AOO) across the 35 sites [29, 30]. Widespread saprotrophs were defined as the ASVs with a representation of > 50% occurrence across all sites, whereas narrow-ranged saprotrophs were defined as having an occupancy of < 10% across all sites. Medium-ranged saprotrophs were defined as the ASVs with occupancy between 20% and 40% across all sites. In summary, ASVs that were defined as widespread saprotrophs distributed across a wider range of environments than narrow-ranged saprotrophs (Supplementary Fig. 3 and 4).

***Community diversity and completeness analyses***

To elucidate the assembly processes underlying biodiversity patterns across geographic gradients in agricultural systems, we focused on community diversity and community completeness. Community diversity was measured as the mean ASV richness (that is, number of saprotrophic fungal phylotypes) per site. We further measured the community completeness using estimates of dark diversity in agricultural fields [31]. Community completeness was calculated as log (community diversity /(dark diversity [32])) at a site. The higher the dark diversity is, the lower is the community completeness. This community completeness reflects how many species from the pool would be expected in a local community. The observed community diversity fails to capture how complete is a local diversity or how many species that could potentially be present (if not limited by dispersal and environmental constraints) are absent from local communities. The number of species actually present and the number of species potentially present determine the completeness of a local community [33]. Patterns of saprotrophic fungal community diversity and completeness were mapped along the distribution range of croplands using the Cubist regression-based spatial interpolation method.

Dark diversity is defined as species that could potentially occupy but are absent at a given site [32]. Dark diversity is unobservable and can only be estimated. Here we used the hypergeometric method that compares the realized number of co-occurrences between pairs of species with random expectations (i.e. no association between species) to estimate dark diversity [34]. The method calculates the mean indication for all absent taxa via constructing a taxon × taxon indication matrix that reflects the probability that taxa occurring in the same samples share similar ecological conditions. The probability for any given taxon is high if the taxa present are positively associated with it, that is if they co-occur more often than expected by chance. To obtain conservative discrete estimates of dark diversity we only kept taxa with greater than 90% probability according to the hypergeometric method [35]. Such a threshold can be considered conservative because probabilities closer to 50% mean either neutrality or noise in the data, whereas higher probabilities suggest that the number of co-occurrences between the absent and present taxa is greater than expected by chance. Given the potential uncertainties inherent in co-occurrence-based estimations [34], particularly in microbial communities from managed soils, dark diversity should be interpreted as a complementary, integrative indicator of community saturation patterns rather than a precise prediction of undetected taxa. Dark diversity was calculated using the “DarkDiv” package in R [34].

***Statistical analyses***

***Pearson correlations***

We used Pearson correlations to determine the associations between geography (latitude and longitude), large-scale factors (MAT, MAP, summer temperature, summer precipitation, LGM MAT, LGM MAP, TA and PA), regional effects, agricultural intensity, soil environment condition, soil environment heterogeneity, biotic interactions (Supplementary Table 1), as well as the community diversity and completeness of widespread, medium, and narrow-ranged saprotrophs. These correlations are displayed with radar charts. We also used Pearson correlations to evaluate the associations between functional genes and enzyme activities related to soil carbon decomposition and the diversity and completeness of saprotrophs with different occupancy, as shown by heatmaps.

***Structural equation modelling***

We used structural equation models to assess potential causal relationships between multiple environmental variables, regional species pool, and the diversity and completeness of saprotrophs with different occupancy across 35 sites. The SEM structure was informed by both ecological theory and the empirical correlation structure of our dataset. Candidate predictors were first examined using Pearson correlations to identify groups of highly correlated variables. All measured variables included in this model were first grouped into ‘composite variables’ and then included in SEMs. Here, we grouped measured environmental variables into ecologically meaningful composite variables based on prior knowledge and correlation structure among variables. These composite variables represent broader environmental gradients (e.g., soil and climate) and were used to reduce model complexity and minimize collinearity in the SEM. We evaluated the robustness of the relationships by reporting the marginal and conditional contributions of environmental predictors to saprotrophic fungal diversity and completeness. Path directions were specified following established ecological understanding of how environmental drivers and local community assembly processes influence regional species pools and local communities. Three metrics were used to quantify the goodness of fit of SEM models: the χ2 test, the root mean square error of approximation (RMSEA), and Comparative Fit Index (CFI). Specifically, the closer to 1 CFI value, closer to 0 RMSEA values, and the higher χ2 and RMSEA P values, the better the model fits. With a good model fit, we were able to interpret the path coefficients of the model and their associated P values. A standardized path coefficient is analogous to the partial correlation coefficient, and describes the strength and sign of the relationship between two variables. These analyses were conducted using the “piecewiseSEM” package [36] in R environment.

***Random forest models***

We used random forest models to determine the ability of diversity and completeness of saprotrophs with different occupancy for predicting soil carbon decomposition. Variable importance was determined by calculating the relative influence of each variable: whether that variable was selected to split the data during the tree building process, and how much the squared error (over all trees) improved (decreased) as a result. Each variable's importance was scaled between 0 and 1. Random forest models were fitted using the h20 package in R.

***Future predictions and mapping***

We used the dataset of the 35 sites to generate maps of likely distributions of widespread saprotrophic fungal diversity. While our study includes 35 sites, these sites comprehensively cover the main climatic and geographic gradients of the study region; therefore, subsequent analyses and projections primarily reflect interpolation within the observed environmental space rather than extrapolation beyond it. First, we conducted ordinary least squares models to project each map under future scenarios (e.g., SSP1-2.6, SSP5-8.5) using environmental predictors derived from CMIP6 projections. According to the most important variables identified to be associated with widespread saprotrophic fungal diversity (Fig. 4a), the model included MAT, MAP, and soil pH. The widespread saprotrophic fungal diversity predicted by the model was positively correlated with diversity obtained from the survey soils (*Pearson* *r* = 0.629, *P* < 0.001). For future projections of the diversity of widespread saprotrophs, we used future climate data from the Inter-Sectoral Impact Model Intercomparison Project phase 3b (ISIMIP3b) project, which provides bias-corrected projections derived from CMIP6 global climate models. These datasets reduce systematic biases in climate simulations and are widely used for ecological impact modeling. We considered three RCPs: SSP1-RCP2.6, SSP3-RCP7 and SSP5-RCP8.5 [37]. The climate created was used as a climate input for the model runs to output widespread saprotrophic fungal diversity under future scenarios.

***Meta-analysis***

To test the general relationships between regional species pool and occupancy of saprotrophic fungi, we collected the ITS amplicon-based sequence data sampled in agricultural systems and surrounding ecosystems using previously published global data. An extensive literature survey was conducted through the Web of Science database (http://apps.webofknowledge.com/) until December 2022. The key words of the literature search were (fung*) AND (land use change OR land cover change OR land use/cover change OR LULCC OR LUCC OR cropland OR farmland OR arable land). A total of 35 publications were collected (Supplementary Fig. 13), comprising 2164 samples of 80 sites (see Source Data). To best avoid the systemic errors derived from sequencing and data processing, saprotrophic fungal communities were investigated by Illumina sequencing. According to the obtained raw abundance value, the same method for identification of saprotrophs with different occupancy was used across 80 sites at global scale.

**References:**

1. Meng Q, Hou P, Wu L *et al.* Understanding production potentials and yield gaps in intensive maize production in China. *Field Crops Res* 2013; **143**: 91-97.

2. Callahan BJ, McMurdie PJ, Rosen MJ *et al.* DADA2: High-resolution sample inference from Illumina amplicon data. *Nat Methods* 2016; **13**: 581-583.

3. Nilsson RH, Larsson K-H, Taylor AF S *et al.* The UNITE database for molecular identification of fungi: handling dark taxa and parallel taxonomic classifications. *Nucleic Acids Res* 2019; **47**: D259-D264.

4. Põlme S, Abarenkov K, Henrik Nilsson R *et al.* FungalTraits: a user-friendly traits database of fungi and fungus-like stramenopiles. *Fungal Divers* 2021; **105**: 1-16.

5. Větrovský T, Kohout P, Kopecký M *et al.* A meta-analysis of global fungal distribution reveals climate-driven patterns. *Nat Commun* 2019; **10**: 5142.

6. Tedersoo L, Bahram M, Polme S *et al.* Global diversity and geography of soil fungi. *Science* 2014; **346**: 1256688.

7. Delgado-Baquerizo M, Bissett A, Eldridge DJ *et al.* Palaeoclimate explains a unique proportion of the global variation in soil bacterial communities. *Nat Ecol Evol* 2017; **1**: 1339-1347.

8. Fordham DA, Saltré F, Brown SC *et al.* Why decadal to century timescale palaeoclimate data are needed to explain present-day patterns of biological diversity and change. *Glob Change Biol* 2018; **24**: 1371-1381.

9. Karger DN, Conrad O, Böhner J *et al.* Climatologies at high resolution for the earth’s land surface areas. *Sci Data* 2017; **4**: 170122.

10. Karger DN, Nobis MP, Normand S *et al.* CHELSA-TraCE21k v1.0. Downscaled transient temperature and precipitation data since the last glacial maximum. *Clim Past Discuss* 2021; **2021**: 1-27.

11. Vasar M, Davison J, Sepp S-K *et al.* Global soil microbiomes: A new frontline of biome-ecology research. *Glob Ecol Biogeogr* 2022; **31**: 1120-1132.

12. Lauber Christian L, Hamady M, Knight R *et al.* Pyrosequencing-Based Assessment of Soil pH as a Predictor of Soil Bacterial Community Structure at the Continental Scale. *Appl Environ Microbiol* 2009; **75**: 5111-5120.

13. Brockett BFT, Prescott CE, Grayston SJ. Soil moisture is the major factor influencing microbial community structure and enzyme activities across seven biogeoclimatic zones in western Canada. *Soil Biol Biochem* 2012; **44**: 9-20.

14. Delgado-Baquerizo M, Reich PB, Khachane AN *et al.* It is elemental: soil nutrient stoichiometry drives bacterial diversity. *Environ Microbiol* 2017; **19**: 1176-1188.

15. Banerjee S, Walder F, Buechi L *et al.* Agricultural intensification reduces microbial network complexity and the abundance of keystone taxa in roots. *ISME J* 2019; **13**: 1722-1736.

16. Tsiafouli MA, Thébault E, Sgardelis SP *et al.* Intensive agriculture reduces soil biodiversity across Europe. *Glob Change Biol* 2015; **21**: 973-985.

17. Zhang M, Wu B, Zeng H *et al.* GCI30: a global dataset of 30 m cropping intensity using multisource remote sensing imagery. *Earth Syst Sci Data* 2021; **13**: 4799-4817.

18. Venter O, Sanderson EW, Magrach A *et al.* Sixteen years of change in the global terrestrial human footprint and implications for biodiversity conservation. *Nat Commun* 2016; **7**: 12558.

19. Wardle DA. The influence of biotic interactions on soil biodiversity. *Ecol Lett* 2006; **9**: 870-886.

20. García-Girón J, Heino J, García-Criado F *et al.* Biotic interactions hold the key to understanding metacommunity organisation. *Ecography* 2020; **43**: 1180-1190.

21. Ma B, Wang Y, Ye S *et al.* Earth microbial co-occurrence network reveals interconnection pattern across microbiomes. *Microbiome* 2020; **8**: 82.

22. Cazelles K, Araújo MB, Mouquet N *et al.* A theory for species co-occurrence in interaction networks. *Theor Ecol* 2016; **9**: 39-48.

23. Gao Q, Yang Y, Feng J *et al.* The spatial scale dependence of diazotrophic and bacterial community assembly in paddy soil. *Glob Ecol Biogeogr* 2019; **28**: 1093-1105.

24. Chen Y, Liu F, Kang L *et al.* Large-scale evidence for microbial response and associated carbon release after permafrost thaw. *Glob Change Biol* 2021; **27**: 3218-3229.

25. Feng J, Wang C, Lei J *et al.* Warming-induced permafrost thaw exacerbates tundra soil carbon decomposition mediated by microbial community. *Microbiome* 2020; **8**: 3.

26. Zheng B, Zhu Y, Sardans J *et al.* QMEC: a tool for high-throughput quantitative assessment of microbial functional potential in C, N, P, and S biogeochemical cycling. *Sci China-Life Sci* 2018; **61**: 1451-1462.

27. Sinsabaugh RL, Follstad Shah JJ. Ecoenzymatic Stoichiometry and Ecological Theory. *Annu Rev Ecol, Evol Syst* 2012; **43**: 313-343.

28. Yuan MM, Guo X, Wu L *et al.* Climate warming enhances microbial network complexity and stability. *Nat Clim Chang* 2021; **11**: 343-348.

29. Staude IR, Pereira HM, Daskalova GN *et al.* Directional turnover towards larger-ranged plants over time and across habitats. *Ecol Lett* 2022; **25**: 466-482.

30. Xu W-B, Blowes SA, Brambilla V *et al.* Regional occupancy increases for widespread species but decreases for narrowly distributed species in metacommunity time series. *Nat Commun* 2023; **14**: 1463.

31. Peng Z, Yang Y, Liu Y *et al.* The neglected roles of adjacent natural ecosystems in maintaining bacterial diversity in agroecosystems. *Glob Change Biol* 2023; **30**: e16996.

32. Pärtel M, Szava-Kovats R, Zobel M. Dark diversity: shedding light on absent species. *Trends Ecol Evol* 2011; **26**: 124-128.

33. Pärtel M, Szava-Kovats R, Zobel M. Community Completeness: Linking Local and Dark Diversity within the Species Pool Concept. *Folia Geobot* 2013; **48**: 307-317.

34. Carmona CP, Pärtel M. Estimating probabilistic site-specific species pools and dark diversity from co-occurrence data. *Glob Ecol Biogeogr* 2021; **30**: 316-326.

35. Trindade DPF, Carmona CP, Reitalu T *et al.* Observed and dark diversity dynamics over millennial time scales: fast life-history traits linked to expansion lags of plants in northern Europe. *Proc R Soc B-Biol Sci* 2023; **290**: 20221904.

36. Lefcheck JS. PIECEWISESEM: Piecewise structural equation modelling in R for ecology, evolution, and systematics. *Methods Ecol Evol* 2016; **7**: 573-579.

37. Hempel S, Frieler K, Warszawski L *et al.* A trend-preserving bias correction – the ISI-MIP approach. *Earth Syst Dynam* 2013; **4**: 219-236.

**Supplementary Table 1.** A complete list of environmental variables and remaining variables used in SEMs for explaining saprotrophic fungal diversity in agricultural systems.

|  | Environmental variables | Abbreviation |
| --- | --- | --- |
| Geographical | Latitude | Latitude |
|  | Longitude | Longitude |
| Large-scale factors | Mean annual temperature since the last glacial maximum | LGM MAT |
|  | Mean annual precipitation since the last glacial maximum | LGM MAP |
|  | Temperature anomaly | TA |
|  | Precipitation anomaly | PA |
|  | Summer temperature | SAT |
|  | Summer precipitation | SAP |
|  | mean annual temperature | MAT |
|  | mean annual precipitation | MAP |
| Regional effects | Regional species pool | Regional species pool |
|  | Natural species diversity | Natural species diversity |
| Agricultural intensity | Human footprint pressure | HFP |
|  | Crop intensity | Crop intensity |
| Biotic interactions | Average degree | ave deg |
|  | Clustering coefficient | clust coeff |
| Soil condition | Soil pH _mean_ | pH |
|  | Organic matter _mean_ | OM |
|  | Available phosphorus _mean_ | AP |
|  | Nitrate nitrogen _mean_ | NO3 |
|  | Ammonia nitrogen _mean_ | NH4 |
|  | Moisture _mean_ | Moisture |
| Soil heterogeneity | Soil pH _sd_ | pH _sd_ |
|  | Organic matter _sd_ | OM _sd_ |
|  | Available phosphorus _sd_ | AP _sd_ |
|  | Nitrate nitrogen _sd_ | NO_3 sd_ |
|  | Ammonia nitrogen _sd_ | NH_4 sd_ |
|  | Moisture _sd_ | Moisture _sd_ |
|  | Plot size | Plot size |

**Supplementary Table 2.** Functional genes information related to carbon degradation.

| Gene name | Encoded protein | Degradation type | Forward sequence (5′ → 3′) | Reverse sequence (5′ → 3′) |
| --- | --- | --- | --- | --- |
| *amyA* | α-amylase | Starch | YGGTTTTCGTCTTGACGCSG | MGGCTGMGTRTCATGRTTK |
| *amyX* | pullulanase | Starch | TATAAYTGGGGMTATGAYCC | CCCATYAAATCAAAWCGRAA |
| *cdh* | cellobiose dehydrogenase | Cellulose | ATWRYCTWCCGMRTHGCCMT | GTKAGSGGRTTBYKGRYCAT |
| *chiA* | endochitinase | Chitin | TSAAGAARTACGCSGACAACG | ASGTCATCAGRCCCTTSAG |
| *glx* | glyoxal oxidase | Lignin | AACCAGTCGATCATCTACGA | RTGSACGAGCTCDGGCATGG |
| *lig* | lignin peroxidase | Lignin | CCGCACACACTGTTGCTGC | CGAAGGATTGCCACTCGCA |
| *pgu* | pectinase/polygalacturonase | Pectin | ANCATTGGTGGCCSTGGAA | TTRAYGGCRATRCARTCRTC |
| *xylA* | xylose isomerase | Hemicellulose | TGGGGBGGTCGYGAAGG | ACTTTGGCRTCRAAGTT |


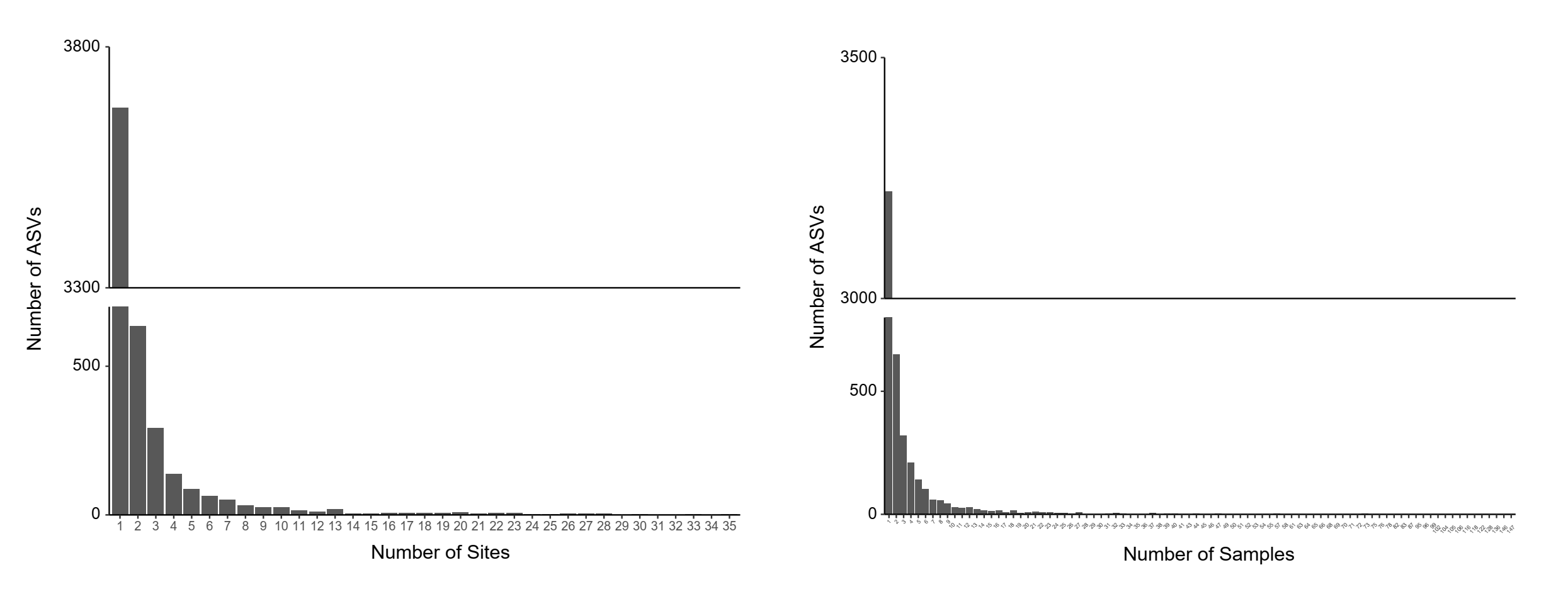


**Supplementary Fig. 1.** Distribution of saprotrophic fungal ASVs in regions and samples.


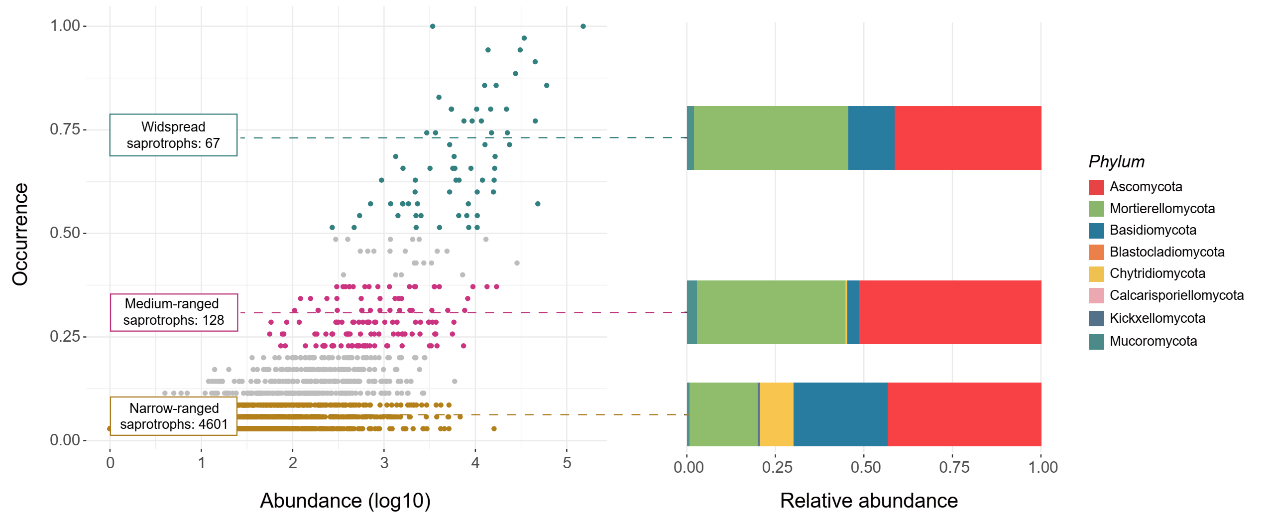
**Supplementary Fig. 2.** Distribution of the different abundance ASVs. The X axis indicates ASVs abundance and the Y axis corresponds to the occurrence (% of regions). Dotted lines delimitate the distributions (the numbers of ASVs of each abundance are displayed in the label) and connect to a box indicating the number of ASVs for each distribution and a bar plot colored by taxonomy at the phylum rank.


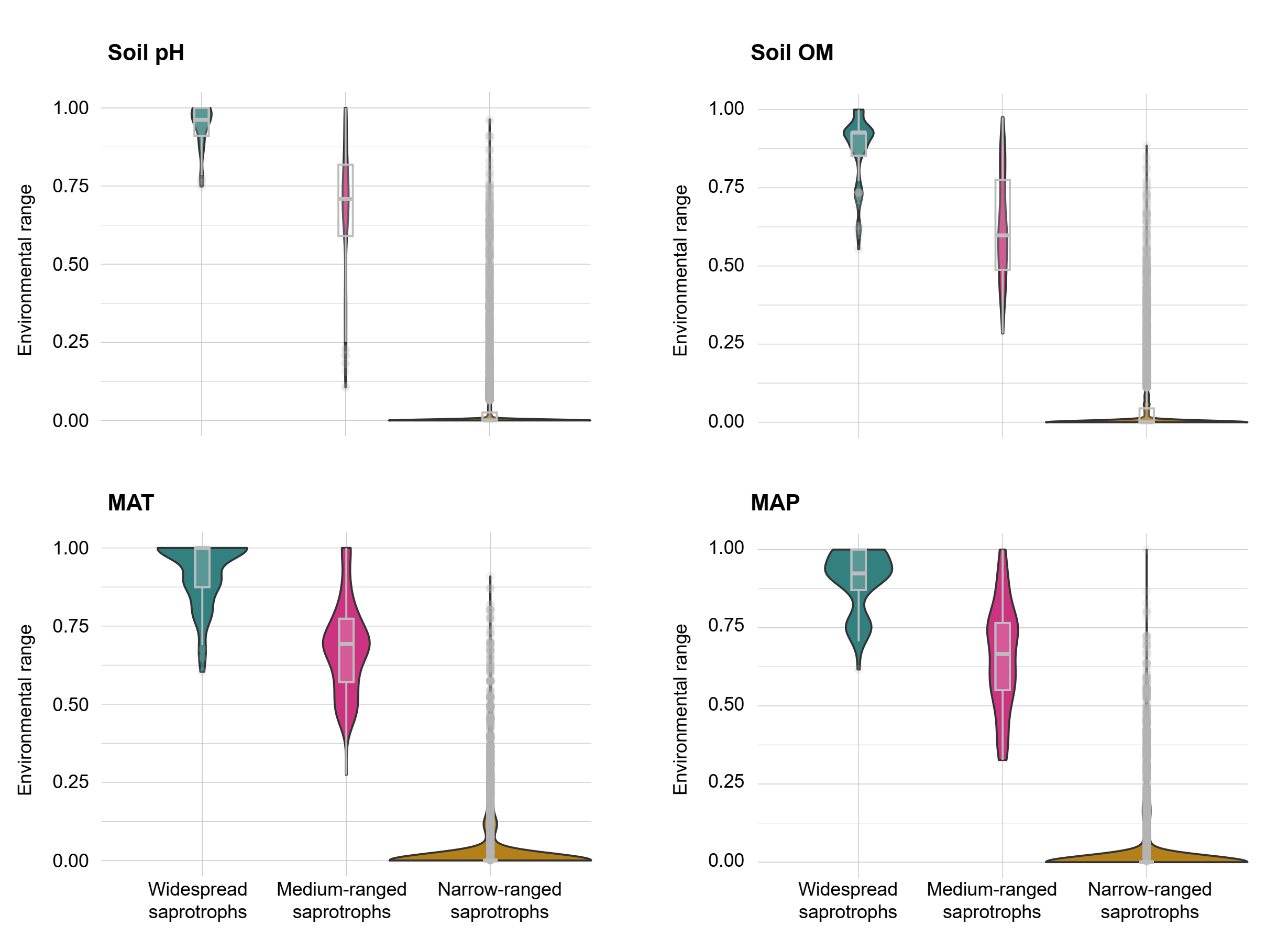


**Supplementary Fig. 3.** Distribution of environmental range (where environmental range is calculated as the average of the range in environmental factors standardized from 0 to 1) for widespread, medium and narrow-ranged saprotrophs. Soil OM, Soil organic matter; MAT, mean annual temperature; MAP, mean annual precipitation.


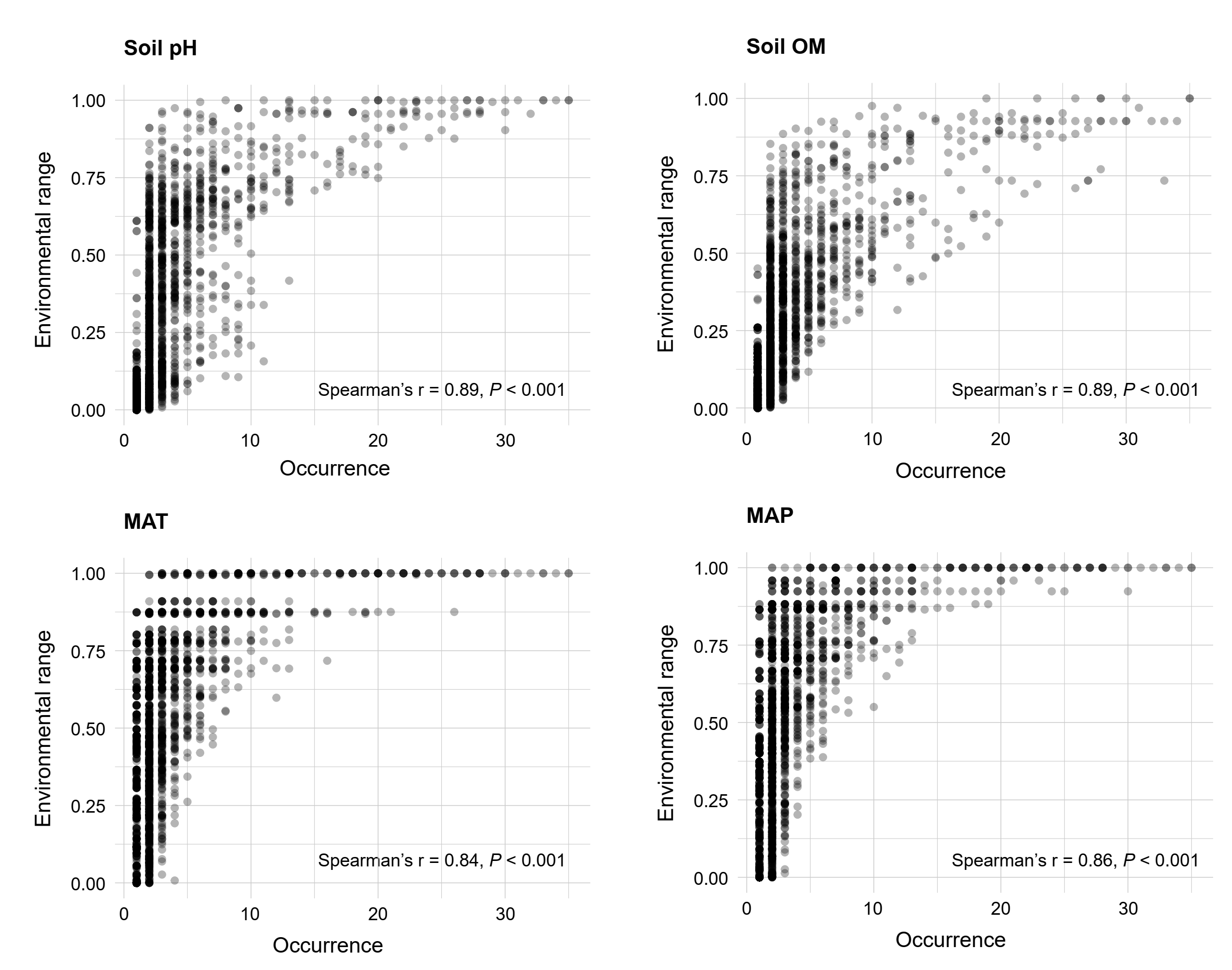


**Supplementary Fig. 4.** Correlation between occurrence and environmental range (where environmental range is calculated as the average of the range in environmental factors standardized from 0 to 1). Soil OM, Soil organic matter; MAT, mean annual temperature; MAP, mean annual precipitation.


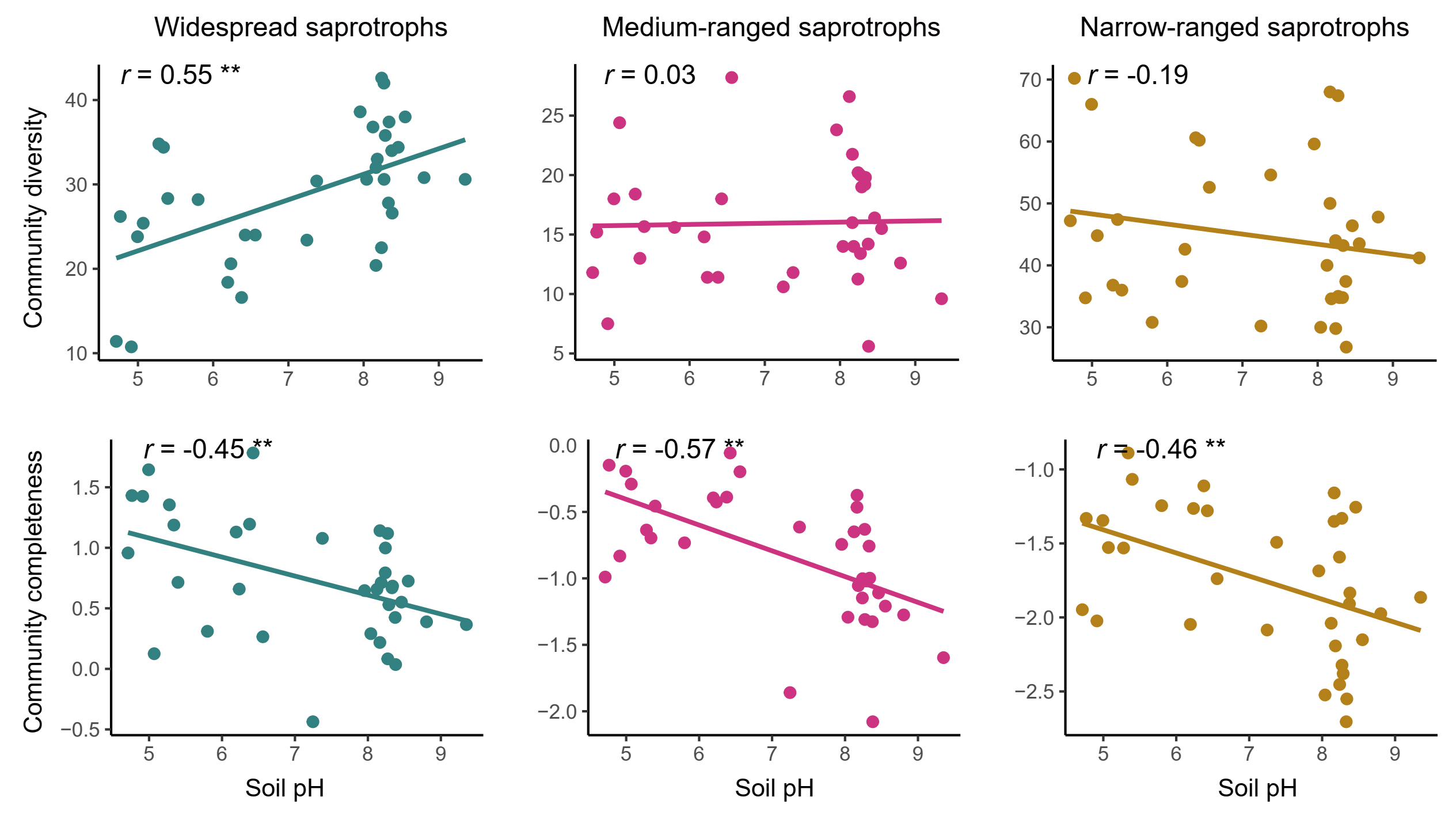


**Supplementary Fig. 5.** Fitted linear relationships between soil pH and the diversity and completeness of different abundance saprotrophs. Pearson correlation coefficients and p values are shown: **p* < .05, ***p* < .01, ****p* < .001.


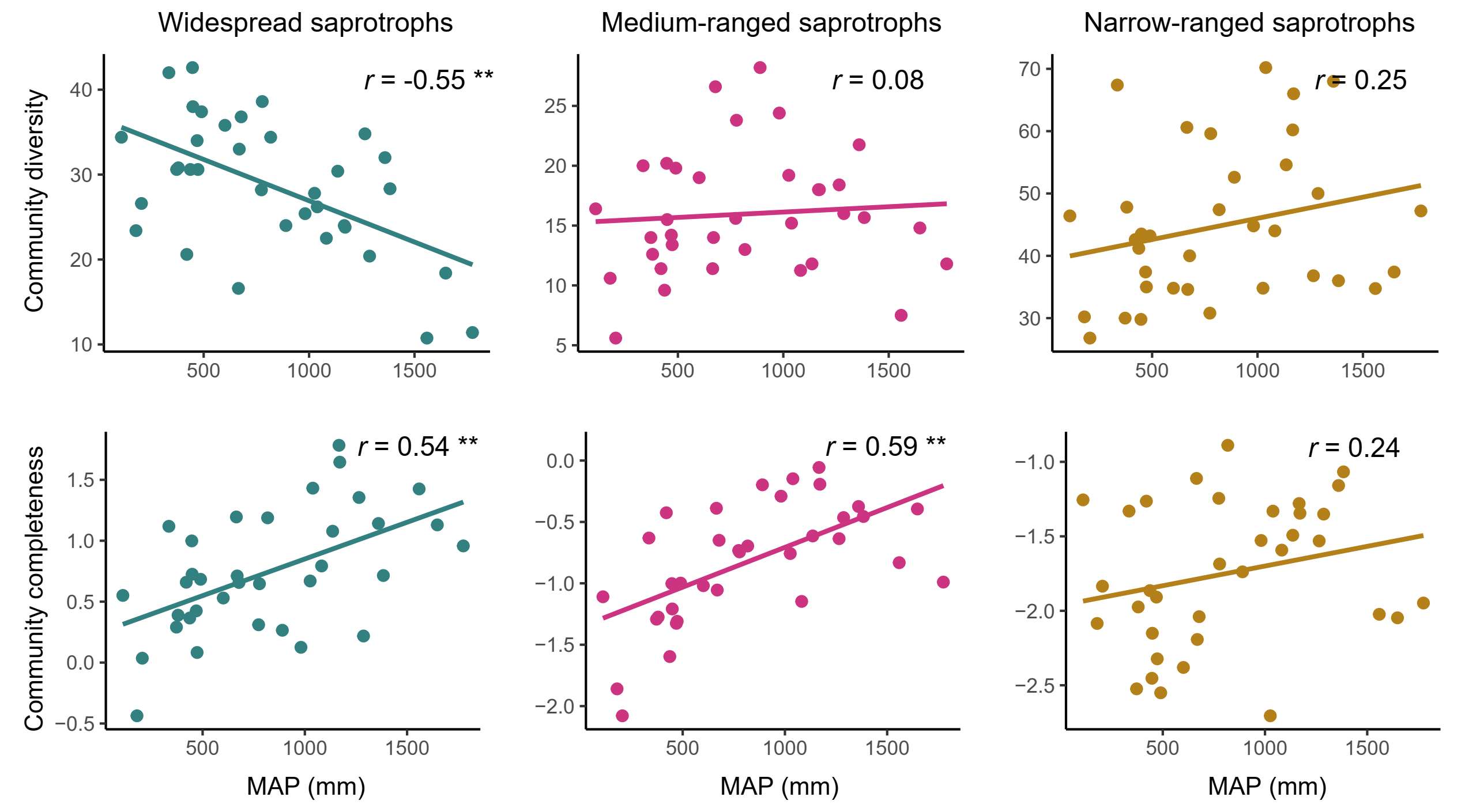


**Supplementary Fig. 6.** Fitted linear relationships between mean annual precipitation (MAP) and the diversity and completeness of different abundance saprotrophs. Pearson correlation coefficients and p values are shown: **p* < .05, ***p* < .01, ****p* < .001.


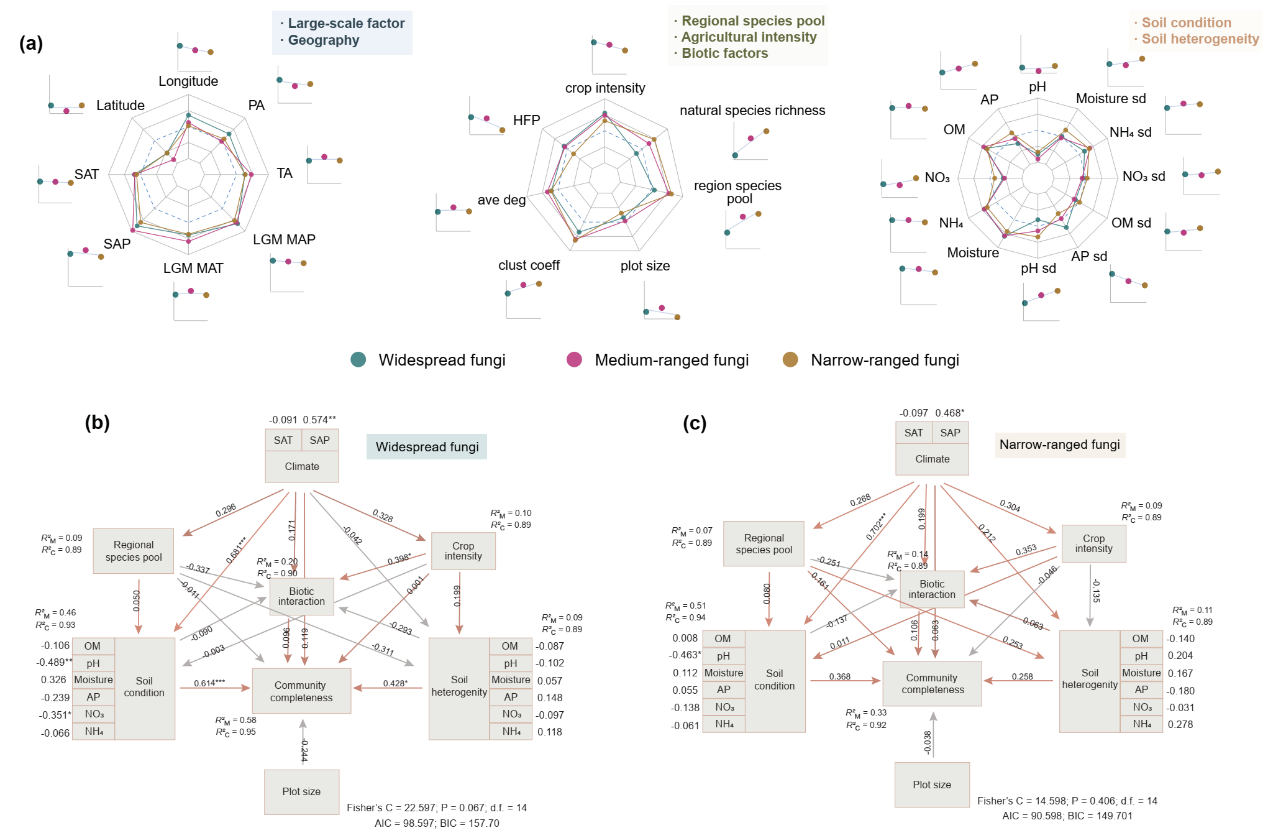
**Supplementary Fig. 7.** Relationships between environmental factors and community completeness of widespread and narrow-range saprotrophs. (**a**) Radar showing the Pearson correlation coefficients between environmental factors and community completeness of widespread (green), medium-ranged (red) and narrow-ranged (yellow) saprotrophs. The first circle from the outermost to the innermost indicates that the correlation coefficient is 1, the second circle indicates 0.5, the third circle (blue dotted lines) is 0, the fourth circle is -0.5, and the fifth circle is -1. The scatterplot and fitted line showing the variation of the correlation coefficient along widespread, medium and narrow taxa. (**b** and **c**) Structural equation models indicating possibly causal relationships between large-scale climate, crop intensity, soil condition, soil heterogeneity, biotic interactions, plot size, the regional species pool, and community completeness of widespread (**b**) and narrow-ranged (**c**) saprotrophs. Red and grey arrows indicate positive and negative relationships, respectively. Numbers adjacent to arrows are path coefficients (partial regression) which represent the directly standardized effect size of the relationship. The conditional (C) and marginal (M) R^2^ represent the proportion of variance explained by all predictors without and with accounting for random effects of “regions”. Significance levels of each predictor are **p* < .05, ***p* < .01, ****p* < .001. TA, Temperature anomaly; PA, Precipitation anomaly; SAT, Summer temperature; SAP, Summer precipitation; LGM MAT, mean annual temperature in the LGM; LGM MAP, mean annual precipitation in the LGM; HFP, human footprint pressure; Ave deg, average degree; Clust coeff, clustering coefficient. A list of environmental factors could be found in Supplementary Table 1.


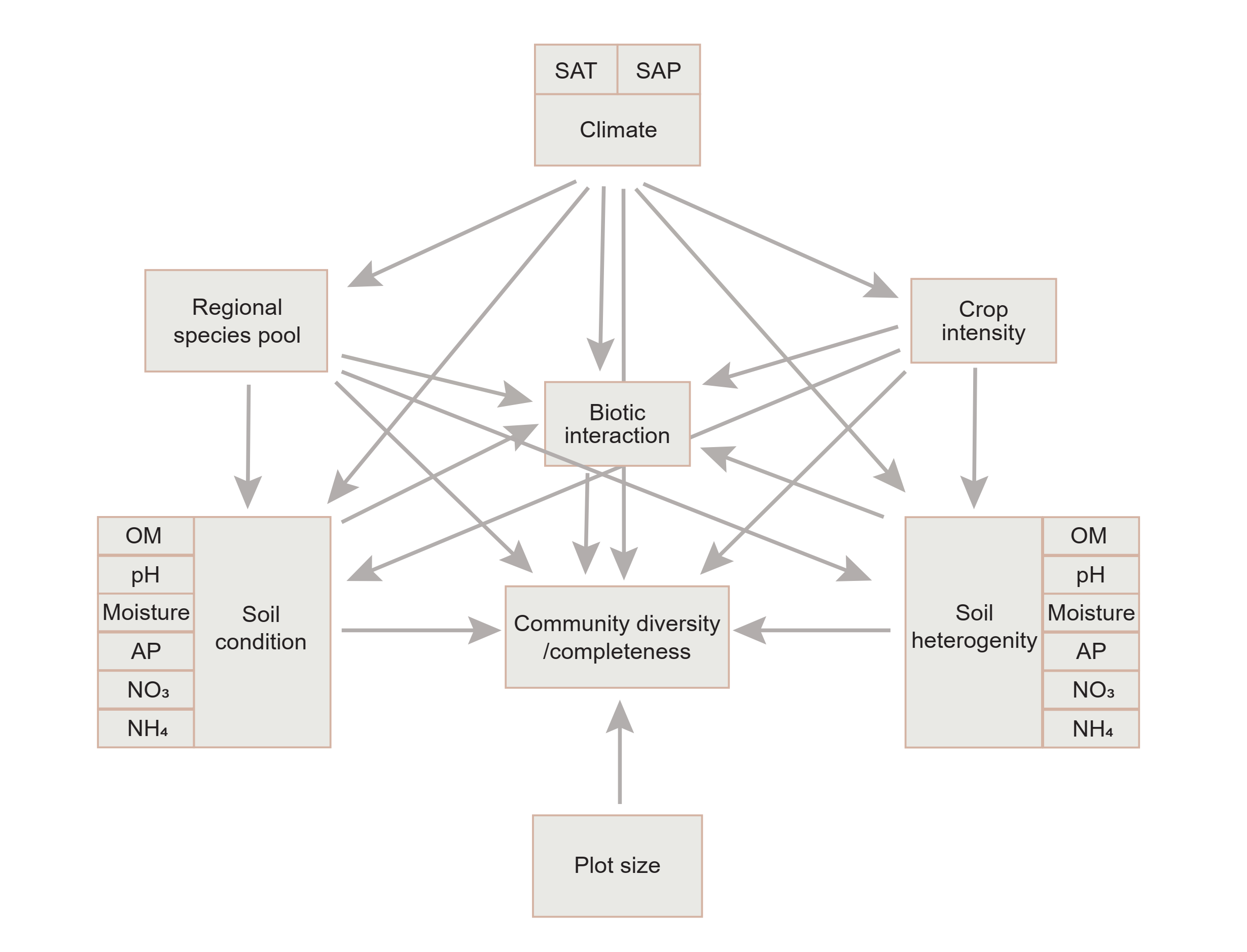


**Supplementary Fig. 8.** A *priori* structural equation model indicating the causal relationships between large-scale climate, crop intensity, soil filtering, soil heterogeneity, biotic interactions, plot size, the regional species pool, and community diversity and completeness of saprotrophs.


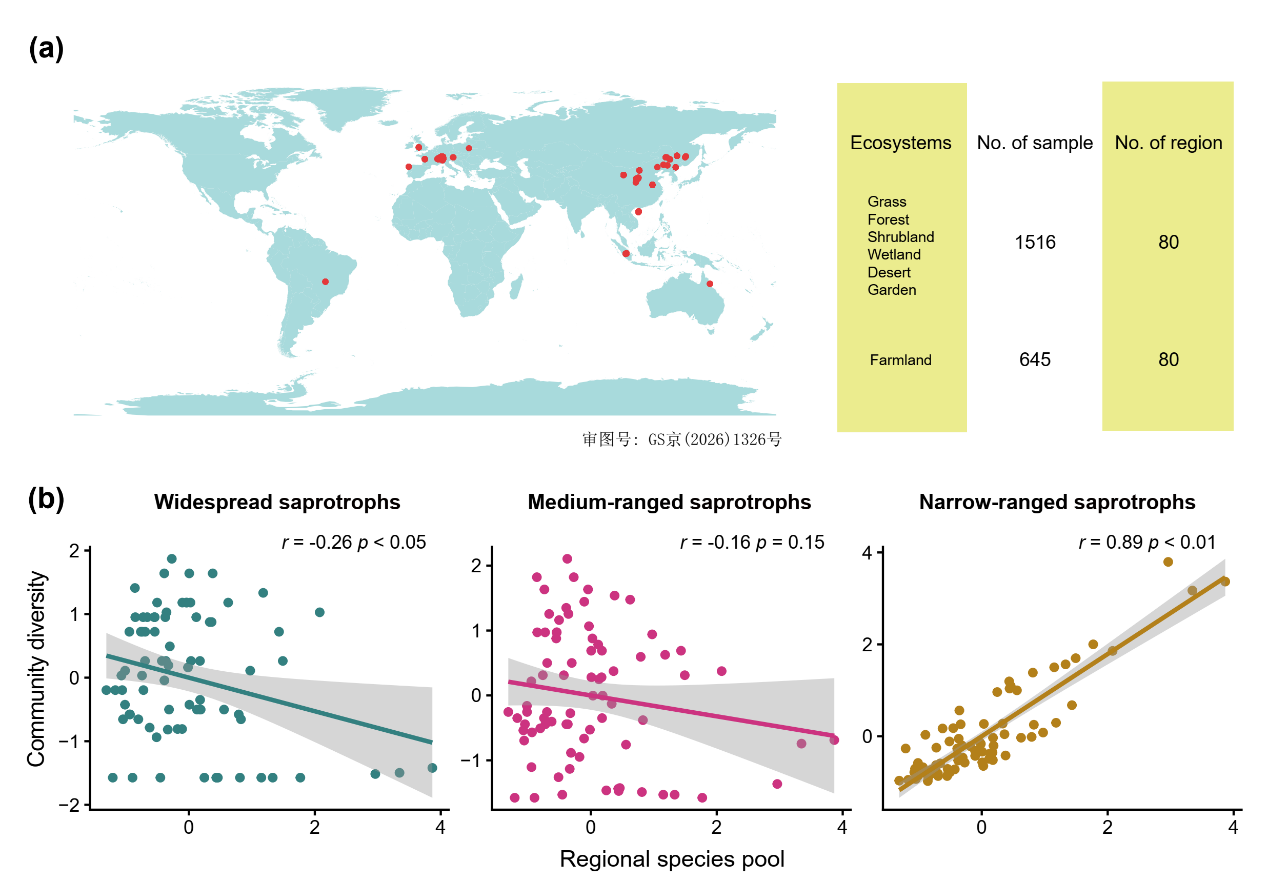
**Supplementary Fig. 9.** Linking regional species pool to community diversity of different abundance saprotrophs at global scale. **(a)** Sampling locations from where data were obtained for fungi from land-use studies. (**b**) Global relationships between regional species pool and community diversity for widespread, medium and narrow-ranged saprotrophs across 80 regions. Lines represent the least squares regression fits and shaded areas represent the 95% confidence intervals. Pearson correlation coefficients and *p* values are shown. Diversity was z-score standardized prior to analysis.


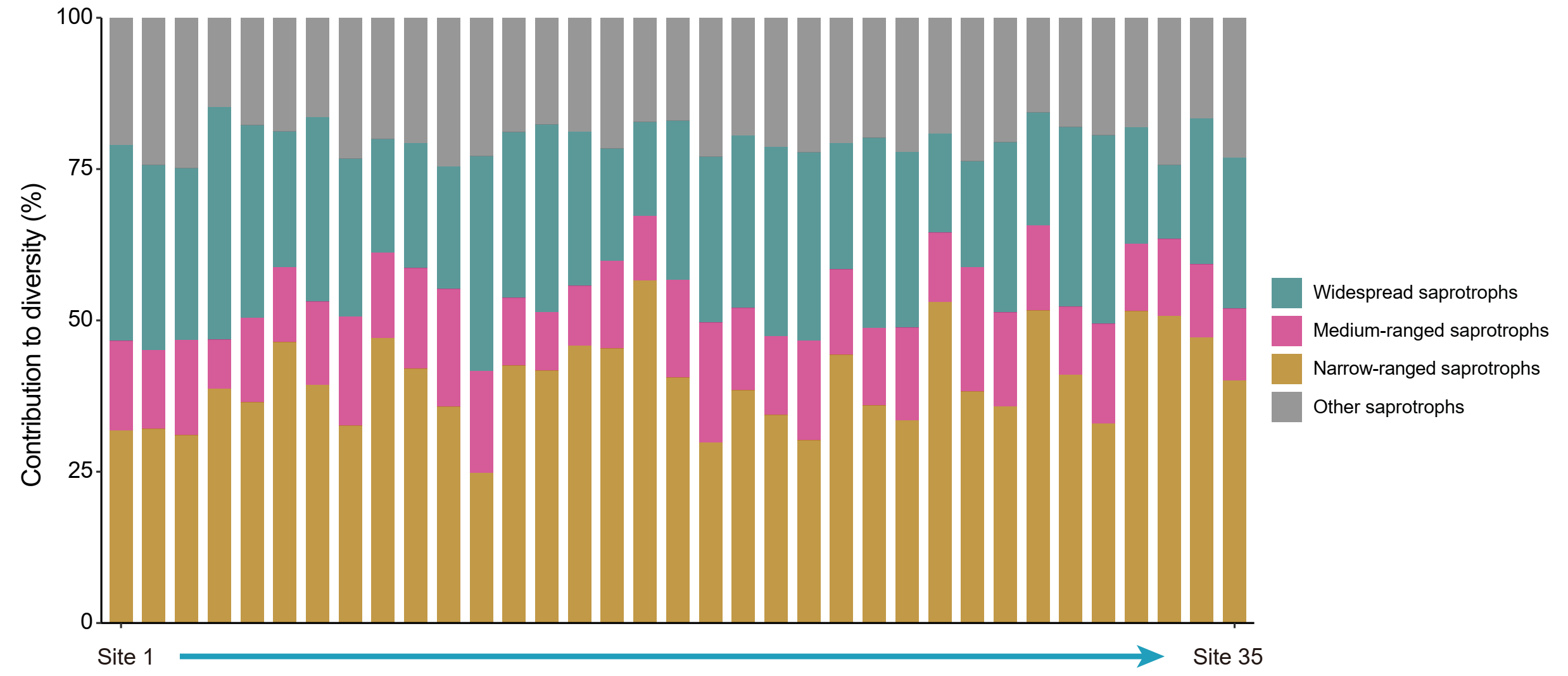


**Supplementary Fig. 10.** Contribution of different abundance of saprotrophic fungal species number to total saprotrophic fungal diversity across 35 regions.

**Supplementary Fig. 11.**
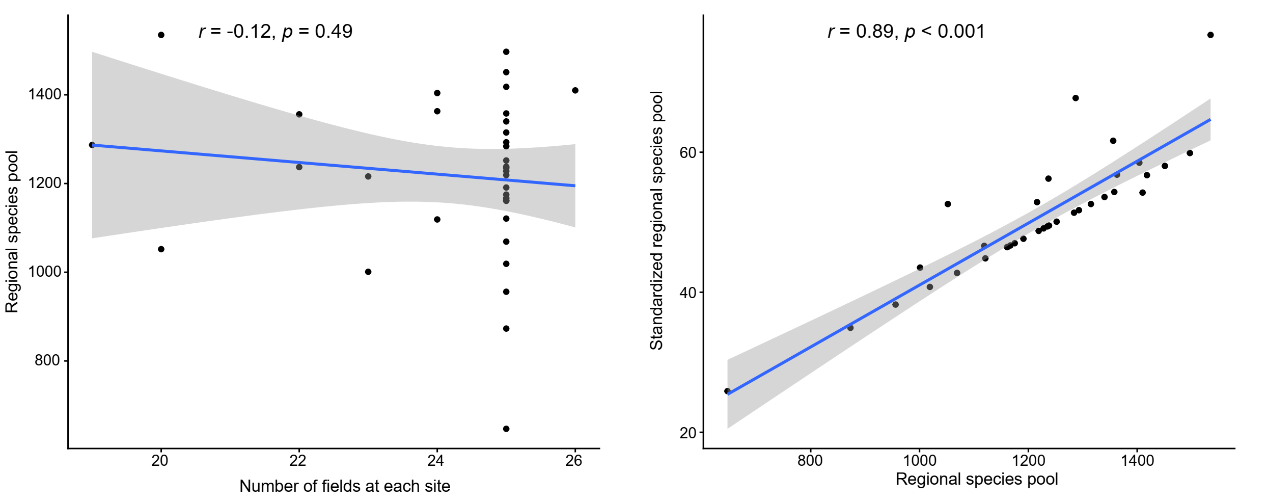
Robustness of regional species pool estimates to sampling effort. (a) Relationship between the number of fields sampled per site and regional species pool richness. Pearson correlation analysis indicates no significant association between the sampling effort (ranging from 19 to 26 fields per site) and the resulting estimates of regional species pool size. (b) Correlation between original and standardized regional species pool richness. A strong positive correlation is observed between the original regional species pool estimates and richness values standardized by the number of sampled fields at each site. This consistency demonstrates that the regional species pool patterns are robust to differences in sampling effort among regions. In both panels, the blue line represents the linear regression fit, and the shaded area denotes the 95% confidence interval.


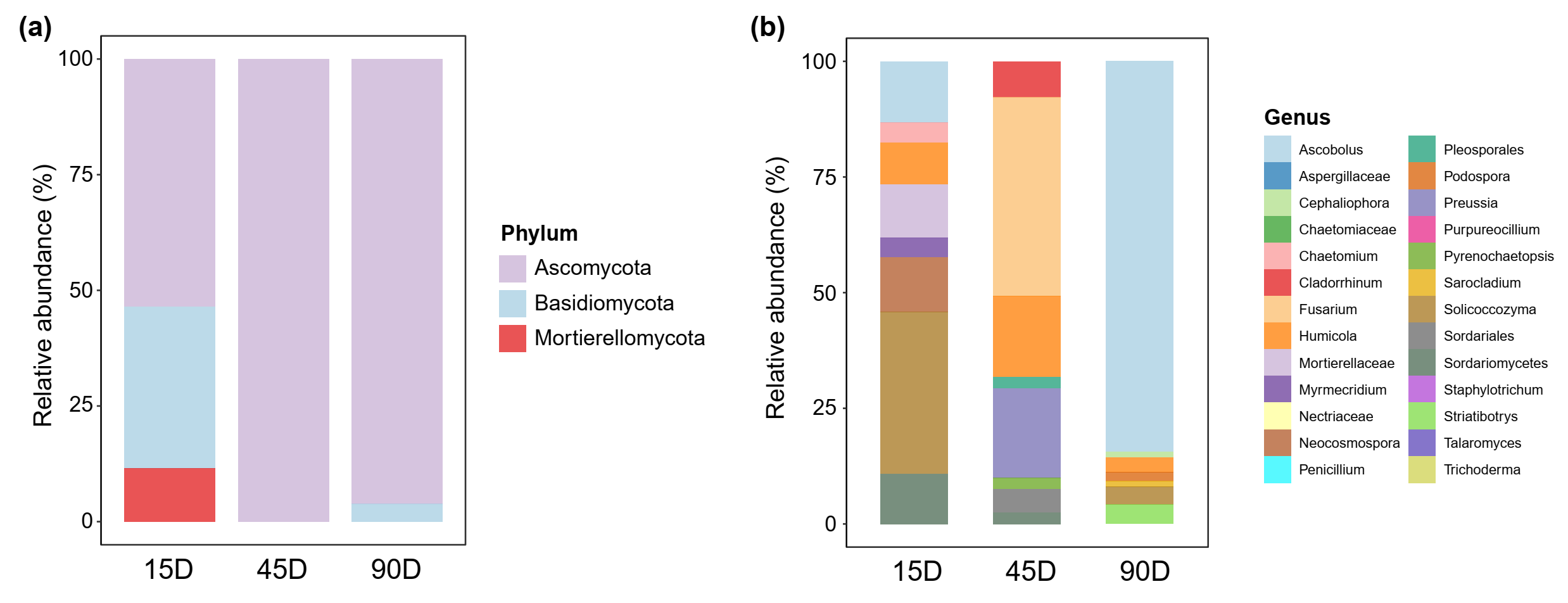
**Supplementary Fig. 12.** Relative phyla and genus abundance of ^13^C-labeled fungal communities. 15D, 15 day; 45D, 45 day; 90D, 90 day.


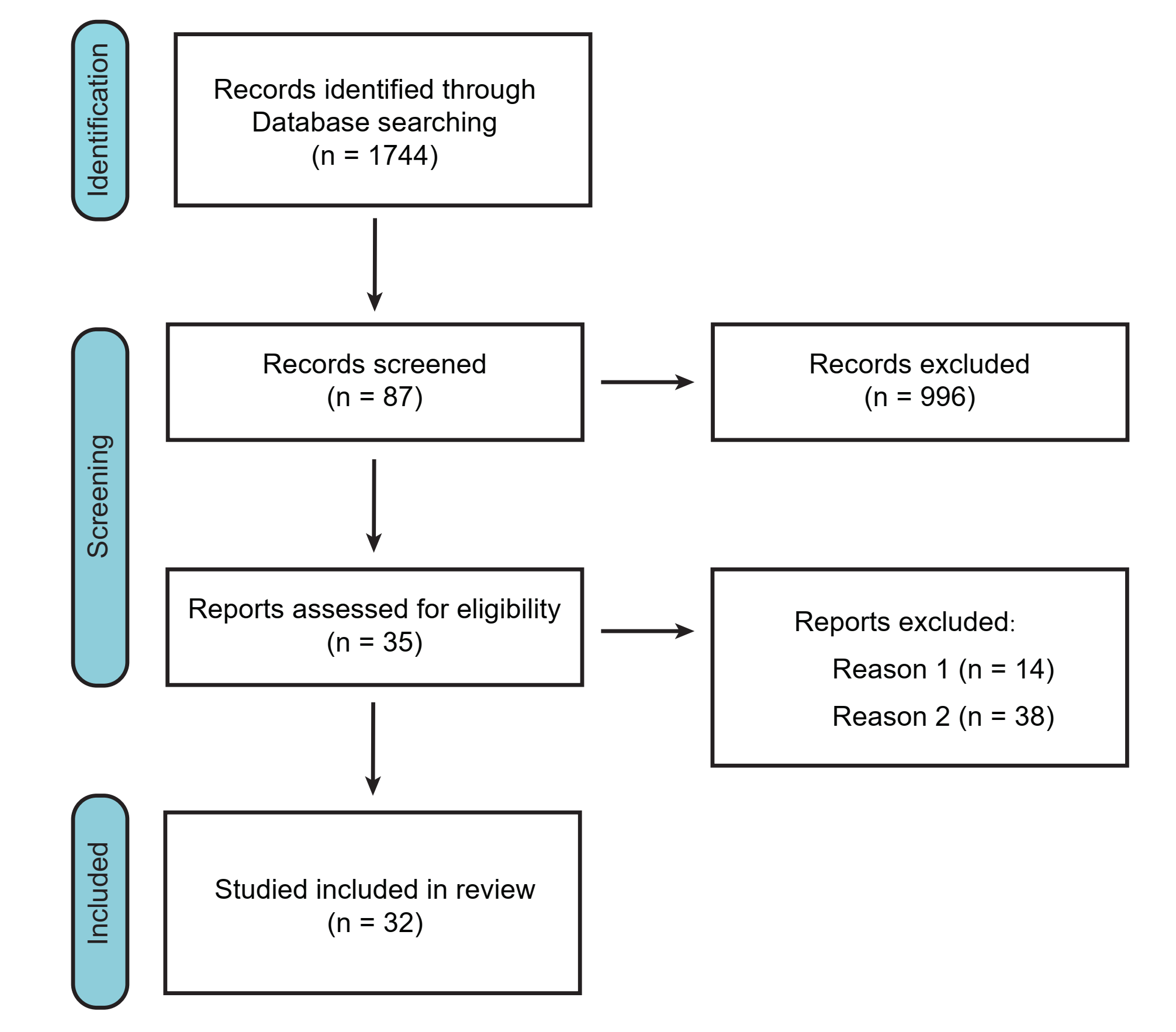


**Supplementary Fig. 13.** PRISMA flow diagram for the studies selected and included in the systematic review.
